# Extraction, Characterization, and Stability Studies of Bistriazinyl-Derived Carboxylic Acids

**DOI:** 10.1021/acs.iecr.5c04766

**Published:** 2026-03-13

**Authors:** Laura Diaz Gomez, Patrik Weßling, Andreas Wilden, Petra J. Panak, Gregory P. Horne, Stephen P. Mezyk, Julie R. Peller, Andreas Geist, Giuseppe Modolo

**Affiliations:** † Forschungszentrum Jülich GmbH, Institute of Fusion Energy and Nuclear Waste Management−Nuclear Waste Management (IFN-2), Jülich 52428 , Germany; ‡ 7891 Heidelberg University, Institute of Physical Chemistry, Im Neuenheimer Feld 253, Heidelberg 69120, Germany; § 150232 Karlsruhe Institute of Technology (KIT), Institute for Nuclear Waste Disposal (INE), P.O. Box 3640, Karlsruhe 76021, Germany; ∥ Center for Radiation Chemistry Research, Idaho National Laboratory, P.O. Box 1625, Idaho Falls ID83415, United States; ⊥ Department of Chemistry and Biochemistry, 14668 California State University Long Beach, 1250 Bellflower Boulevard, Long Beach, California 90840-9507, United States; # Department of Chemistry, Valparaiso University, 1710 Chapel Drive, Valparaiso, Indiana 46383, United States

## Abstract

Separation of An­(III) and Ln­(III) ions will benefit the recycling of used nuclear fuel (UNF). For this purpose, many ligands have been tested over the years, and several separation processes have been successfully demonstrated on the laboratory scale. Current research aims at the development of new ligands that are built only with carbon, hydrogen, oxygen, and nitrogen (CHON), as they can be incinerated completely without secondary waste production. Here, we tested a new class of water-soluble ligands, the bistriazinyl-octa-carboxylic acids. One member, in particular, 2,6-bis-[5,6-di­(3,4-dicarboxyphenyl)-1,2,4-triazin-3-yl]-pyridine (BTPOA), was found to be suitable for the selective separation of Am­(III) and Cm­(III) ions from Ln­(III) ions and may act as a CHON alternative to its sulfonated analogue (SO_3_-Ph-BTP). BTPOA exhibited good extraction results and a high selectivity for Am­(III) over Eu­(III) ions. This ligand’s complexation of metal ions was further studied using potentiometric spectroscopy, as well as time-resolved laser-induced spectroscopy with Cm­(III) and Eu­(III) in aqueous HClO_4_ and HNO_3_ media. Conditional stability constants of each formed species were determined. In the HClO_4_ system, Cm­(III) formed three species (1:1, 1:2, and 1:3) through the stepwise addition of a single BTPOA molecule. On the other hand, in HNO_3_, Cm­(III) formed two 1:2 complexes and one 1:3 complex, while the stepwise formation of three species was observed for Eu­(III). The stability constants are comparable to the values for SO_3_-Ph-BTP. The radiolytic behavior of BTPOA was also investigated using electron pulse irradiation measurements to determine absolute rate coefficients (*k*) under ambient temperature conditions for the reaction of BTPOA with typical UNF reprocessing radical radiolysis products—the hydrated electron (e_aq_^–^, *k* = (1.60 ± 0.02) × 10^10^ M^–1^ s^–1^), the hydrogen atom (H^•^, *k* = (2.17 ± 0.03) × 10^9^ M^–1^ s^–1^), and hydroxyl (^•^OH, *k* = (6.95 ± 0.06) × 10^9^ M^–1^ s^–1^) and nitrate (NO_3_^•^, *k* = (0.37 ± 0.02) × 10^7^ M^–1^ s^–1^) radicals. These rate coefficients indicate that the radiolytic longevity of BTPOA should increase with HNO_3_ concentration, owing to the consumption of e_aq_^–^/H^•^, by nitrate anions, and the replacement of ^•^OH by the less reactive NO_3_^•^.

## Graphical abstract

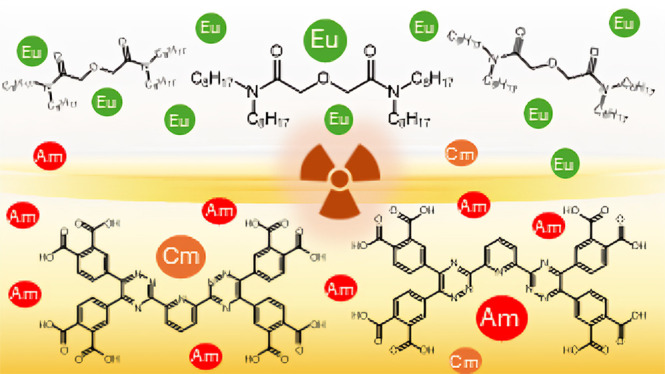

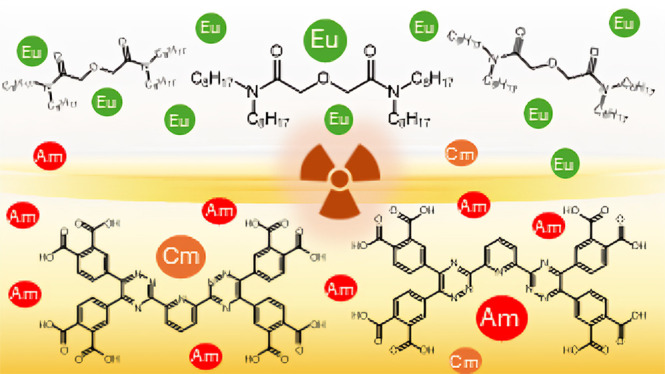

## Introduction

The separation of minor actinides (An), lanthanides (Ln), and other fission products from used nuclear fuel (UNF) or plutonium uranium reduction extraction (PUREX)^1^ raffinate will benefit nuclear waste management strategies by reducing the radiotoxicity and decay heat burden on long-term storage.^2,3^ Am­(III), Cm­(III), and Ln­(III) ion separation is a particularly challenging task due to their similar atomic radii and chemical properties. In the last two decades, different studies were published on the separation of An­(III) from Ln­(III) (GANEX and EURO-GANEX),^4–6^ the coseparation of Am­(III)/Cm­(III) from Ln­(III) (SANEX and *i*-SANEX),^7–11^ and the selective separation of Am­(III) from UNF (AmSel and EXAm).^12,13^ These processes use solvent extraction techniques to selectively complex metal ions of interest using organic and/or water-soluble ligands.

The bistriazinyl (BTP) family are N-donor ligands that are highly selective toward An­(III) over Ln­(III). They have been studied as lipophilic ligands^12,14–17^ and can also be modified to be hydrophilic masking agents/hold-back reagents by inclusion of sulfophenyl, hydroxyl, carboxylic acid, or other hydrophilic functional groups to the outer periphery of the BTP architecture.^14^ Their structures have been refined to increase their stability in acidic media, improve selectivity, and create a synergistic extraction system by combining lipophilic and hydrophilic ligands with inverse selectivity.^18^

For example, the *i*-SANEX^8^ solvent system uses *N*,*N*,*N′*,*N′*-tetraoctyl diglycolamide (TODGA, Figure 1) in combination with SO_3_-Ph-BTP^18^ to produce the desired synergistic system.^14,16^ The hydrophilic SO_3_-Ph-BTP ligand complexes retain Am­(III) and Cm­(III) in the aqueous phase, allowing for the extraction of Eu­(III) and other Ln­(III) TODGA complexes into the organic phase. Ligands with only carbon, hydrogen, oxygen, and nitrogen in their structure (so-called CHON principle) are currently the preferred option because they can be fully incinerated without further treatment.^19^ Several studies have tested a variety of CHON molecules to achieve these separation objectives.^20–22^ The present study focuses on a CHON alternative to SO_3_-Ph-BTP, substituting the sulfophenyl groups for carboxylic acid groups (BTPOA, Figure 1), although the impact of this substitution on separations is currently unknown.

**Figure 1:**
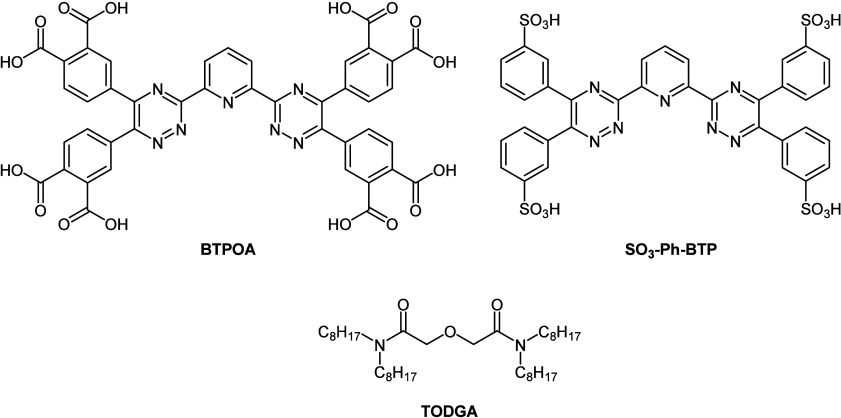
Molecular structures of BTPOA, SO_3_-Ph-BTP, and TODGA.

In addition to good solubility in aqueous acidic media, another key aspect for candidate ligands under UNF reprocessing conditions is their longevity in the presence of ionizing radiation fields. Irradiation of reprocessing solvent systems typically results in the destruction of ligands and the formation of problematic degradation products, the consequences of which determine the An­(III)/Ln­(III) separation efficiency and solvent recyclability. The radiation chemistry of BTP molecules has been investigated in several process-relevant media, although the majority of these studies focused upon the organic phase.^23–36^ In aqueous solutions, the radiolytic behavior of SO_3_-Ph-BTP has been investigated.^37,38^ Using a combination of experiment and multiscale modeling methods, Horne et al.^39^ provided a thorough characterization of SO_3_-Ph-BTP radiolysis in neat water, finding near-complete (>90%) ligand destruction within 1 kGy of absorbed gamma radiation dose, for which the oxidizing hydroxyl radical (^•^OH, *E*° = 2.7 V)^40^ was shown to be predominantly responsible. Galan^37^ and Peterman^38^ reported greater SO_3_-Ph-BTP radiolytic longevity (100s of kGy) in dilute nitric acid (HNO_3_) media. Although both studies indirectly probed the extent and impacts of SO_3_-Ph-BTP radiolysis using An­(III)/Ln­(III) distribution ratios, a basis for enhanced SO_3_-Ph-BTP longevity in HNO_3_ solution could be argued in terms of changes in the suite of available radiolysis products. More specifically, as the concentration of HNO_3_ is increased, neat water radiolysis products (eq 1)^40^ are progressively converted into HNO_3_ radiolysis products (eqs 2–7):^40–46^

H2O⇝eaq−,H•,OH•,H2O2,H2,Haq+ 1

$${\mathrm{HNO}}_{3}/{{\mathrm{NO}}_{3}}^{-}⇝{e_{\mathrm{aq}}}^{-},{{\mathrm{NO}}_{3}}^{•},{\mathrm{HNO}}_{2}/{{\mathrm{NO}}_{2}}^{-},O^{•},{H_{\mathrm{aq}}}^{+}$$ 2

$$\begin{array}{cc} {\mathrm{HNO}}_{3}⇌{{\mathrm{NO}}_{3}}^{-}+{H_{\mathrm{aq}}}^{+} & \mathrm{pKa}∼-1.37 \end{array}$$ 3

$${e_{\mathrm{aq}}}^{-}+{{\mathrm{NO}}_{3}}^{-}\to {{\mathrm{NO}}_{3}}^{•2-}\overset{2{\mathrm{Haq}}^{+}}{\to }(H_{2}O+{{\mathrm{NO}}_{2}}^{•})\to \to {\mathrm{HNO}}_{2},k=9.7\times 10^{9}M^{-1}s^{-1}$$ 4

$$H^{•}+{{\mathrm{NO}}_{3}}^{-}\overset{{\mathrm{Haq}}^{+}}{\to }(H_{2}O+{{\mathrm{NO}}_{2}}^{•})\to \to {\mathrm{HNO}}_{2},k=5.6\times 10^{6}\;M^{-1}s^{-1}$$ 5

$$\begin{array}{cc} {\mathrm{OH}}^{•}+{\mathrm{HNO}}_{3}\to {{\mathrm{NO}}_{3}}^{•}+H_{2}O & k=5.3\times 10^{7}\;M^{-1}s^{-1} \end{array}$$ 6

$$\begin{array}{cc} H_{2}O_{2}+{\mathrm{HNO}}_{2}\to {{\mathrm{NO}}_{3}}^{•}+H_{2}O & k=1.9\times 10^{7}\;M^{-1}s^{-1} \end{array}$$ 7

Most notably, the ^•^OH is replaced by the typically less oxidizing nitrate radical (NO_3_^•^, *E*° = 2.3–2.6 V).^47^ Given these changes, a greater radiolytic longevity may be expected for SO_3_-Ph-BTP in HNO_3_ media. That said, Kynman et al. recently showed that the radiation-induced chemical reactivity of SO_3_-Ph-BTP is influenced by actinide complexation, reporting an order-of-magnitude increase in its rate of reaction with NO_3_^•^ when complexed to Am­(III).^48^

Irrespective, substitution of the solubilizing sulfophenyl functionality in SO_3_-Ph-BTP for carboxylate groups will ultimately alter the BTP molecule’s radiolytic behavior and, thus, its longevity under envisioned process conditions. Consequently, the interaction of BTPOA with typical UNF reprocessing radical radiolysis products—the hydrated electron (e_aq_^–^), the hydrogen atom (H^•^), ^•^OH, and NO_3_^•^—must be quantitatively understood to evaluate the feasibility of this ligand under real-world process conditions.

Considering the above, our goal is to evaluate the separations and radiolytic stability properties of BTPOA to assess its suitability as a replacement for SO_3_-Ph-BTP in the *i*-SANEX or GANEX process solvent system. Here, we report stability studies for hydrolysis, radiation-induced reaction kinetics, and distribution ratio temperature dependence, as well as characterization studies, including kinetics, acid constant (p*K*_a_) determination, and the speciation of BTPOA complexes of An­(III) and Ln­(III) via time-resolved laser-induced fluorescence spectroscopy (TRLFS).^49^

## Methods

### Ligand Synthesis

The company is unable to disclose the synthesis route. Nevertheless, it is imperative to emphasize that the ligand was synthesized with eight carboxylic acids in the outer sphere with the objective of enhancing solubility and, consequently, the solubility factor. This is predicated on the fact that only four acids will be feasible in the salt form.

### Solvent Extraction

Batch solvent extraction experiments were carried out using equal volumes of 500 μL of each phase. The aqueous and organic phases were pipetted into screw-cap glass vials and contacted for a given time on an IKA VIBRAX VXR, IKA-Werke GmbH & Co. KG (Staufen, Germany) basic automatic shaker at 2,200 rpm and 22 °C. The temperature was controlled by a F25-HE thermostat, JULABO GmbH (Seelbach, Germany). After mixing, the samples were centrifuged with a Hettich EBA 8s centrifuge, Andreas Hettich GmbH & Co.KG (Tuttlingen, Germany), for 5 min. The phases were then separated manually using a fine-tipped transfer micropipette. 200 μL of each phase was transferred into new glass vials for further analyses.

Gamma measurements of ^241^Am (60 keV) and ^152^Eu (122 keV) were carried out using an Eurisys EGC 35–195-R germanium coaxial N-type detector, and spectra were evaluated using the GammaVision Software. Samples were measured directly without further treatment. Alpha measurements were carried out for ^241^Am (5486 keV) and ^244^Cm (5805 keV) using an Ortec/Ametek ALPHA-ENSEMBLE-8 eight-chamber alpha measurement system equipped with PIPS detectors purchased from Ametek GmbH (Meerbusch, Germany). Sample preparation for alpha measurement was done by homogenizing a 10 μL alpha-spectroscopy sample in 100 μL of a mixture of Zapon varnish and acetone (1:100 v/v). This mixture was distributed over a stainless-steel plate obtained from Berthold, Bad Wildbad, Germany. The sample was dried under a heating lamp and annealed into a stainless-steel plate by a gas-flame burner. For stable elements, inductively coupled plasma mass spectrometry (ICP-MS) was applied using a PerkinElmer NexION 2000C. Aqueous samples were measured after dilution in 1% v/v HNO_3_ solution without further treatment. Organic samples were measured directly in a surfactant matrix (Triton-X-100) in 0.1% v/v HNO_3_ after dilution and were compared with back extraction with 0.5 M ammonium glycolate in 1% HNO_3_.

### Simulated High Active Raffinate (HAR)

To process the simulated PUREX raffinate solution with the proposed system, a series of steps must be carried out in a specific order. First, the feed solution is mixed with 1,2-diaminocyclohexane-*N*,*N*,*N′*,*N′*-tetracetic acid (CDTA) until a concentration of 50 mM is reached, acting as a masking agent for Pd and Zr.^1^ Subsequently, the loading step is conducted by mixing the HAR + CDTA solution with 0.2 M TODGA with the objective of transferring the trivalent actinides and lanthanides to the organic phase. Afterward, the organic phase must be cleaned of any unwanted coextracted metals, a process known as scrubbing. This is achieved by mixing the loaded organic phase with 0.5 M HNO_3_. This step can be repeated as necessary; however, in this case, it was performed once. Finally, the stripping step took place by combining the loaded TODGA with the aqueous phase, which contained 0.01 M BTPOA in different concentrations of HNO_3_ and 10 μM of all Ln (except *Pm*).

Distribution ratios (*D*) were calculated as the ratio of activity or metal ion (M) concentration in the organic phase vs the activity or metal ion concentration in the aqueous phase ([M]_org_/[M]_aq_). The separation factor (SF) between two metal ions was calculated as the ratio of the corresponding distribution ratios (SF_M1/M2_ = *D*_M1_/*D*_M2_). Distribution ratios between 0.01 and 100 exhibit an uncertainty of ±5%, while lower/higher values exhibit larger uncertainties. Mass balances were calculated as the sum of the aqueous and organic concentrations divided by the initial concentration.

### p*K*_a_ Determination

First, the titrator TITRANDO was calibrated by titrating 0.1 M oxalic acid with 0.1 M NaOH and 0.1 M HNO_3_. Afterward, we proceeded with electrode calibration with buffer solutions of pH 1, 4, 7, 10, and 12. Known concentrations of HNO_3_ and NaOH were tested. Solutions with 8 mg of BTPOA, 30 mg of BTBPOA, and 30 mg BTPhenOA were prepared and titrated with 0.1 M NaOH and 0.1 M HNO_3_ to obtain the deprotonated and protonated forms of the molecules.

Another approach to keep the ionic strength constant consisted of adding NaNO_3_ into the titrant 0.1 M NaOH. In addition, the sample was diluted in a solution of 0.1 M HNO_3_ and 1.9 M NaNO_3_ to have a final nitrate concentration of 2 M. The first trial started with a 5 mM ligand solution at pH 1 to reach the full protonation of the ligand and to stepwise deprotonate it with the titrant. The second trial, the titrant was changed to 1 M NaOH with 1 M NaNO_3_, and the concentration of the ligand was decreased to 2.5 mM, and for the last trial, the concentration of the ligand increased to 7.5 mM. The data was analyzed using *Hyperquad 2013*.

### TRLFS Titration

For these titration experiments, monophasic samples of Cm­(III) and Eu­(III) in the respective acidic media were prepared. For Cm­(III) and Eu­(III), 4.7 μL of 21.2 μM Cm­(ClO_4_)_3_ and 9.4 μL of 1.07 mM Eu­(ClO_4_)_3_ were dissolved in 1 mL of 1 mM HClO_4_, resulting in stock solutions of 0.1 μM Cm and 10 μM Eu. First scoping experiments showed that the Eu concentration was too high, resulting in an overflow of the camera due to the high fluorescence intensity. Therefore, in the case of Eu, it was necessary to reduce the concentration by 2 orders of magnitude, resulting in a stock solution concentration of 0.1 μM. Aliquots of ligand solution were added stepwise, and the emission spectra were recorded after reaching chemical equilibrium for each step. For the Cm-nitrate system, the laser energy was reduced with the objective of avoiding any compromise to the structure of the ligand or complex.

## Results and Discussion

### Solubility

The initial step in characterizing these compounds and assessing their potential use in solvent extraction is to evaluate their solubility in aqueous HNO_3_, as this is used as the aqueous phase in the conventional PUREX process. All three ligands are soluble in water at concentrations of up to 0.1 M. In HNO_3_, their solubility decreases as the concentration of acid increases. BTPOA has been demonstrated to remain soluble in acid media even at high concentrations (≥5 M for 10 mM BTPOA solution).

Solubility is a key property in selecting the ligands for subsequent experiments. In addition to aqueous solubility, the maximum HNO_3_ concentration at which each ligand remains soluble was considered, as the ligands are used in combination with an organic extractant. Under these conditions, TODGA is ineffective at relatively low HNO_3_ concentrations. Consequently, BTPOA is the only suitable water-soluble ligand for solvent extraction and further investigations.

### Acid Constant Determination

The BTPOA p*K*_a_ value was determined through potentiometric titration and validated through the *Hyperquad* software. The potentiometric titration was performed with NaOH and HNO_3_ measuring only a single equivalent point across all trials. The outcome differed from the expected range of eight points, suggesting the possibility of overlapping or undetectable equivalence points. To ascertain the single equivalence point, we employed the first derivative, calculating a value through the Henderson–Hasselbalch (eq 8), establishing an average p*K*_a_ value of 4.63 ± 0.05, with HA denoting the acid and A^*–*^ denoting the conjugate base of the ligand:

$$-\log[H_{3}O^{+}]=-\log[K_{a}]+\log\frac{\left[A^{-}\right]}{\left[\mathrm{HA}\right]}$$ 8

To corroborate the obtained p*K*_a_ through *Hyperquad*, we performed a titration with controlled ionic strength, fitting the experimental data in the program. This yielded four p*K*_a_ values, all of which were similar to those previously calculated.

The calculated p*K*_a_ value corresponds to those of the carboxylic acid family (∼5), which would be expected for BTPOA. However, it is necessary to consider that the ligand can also be protonated at the pyridine nitrogen of the central ring in addition to the eight carboxylic groups in the periphery. The lowest pH value that could be measured with the available equipment was 0.99, which is above the p*K*_a_ for the pyridine nitrogen of 0.5 calculated for SO_3_-Ph-BTP.^50^ Therefore, the results obtained correspond to the eight carboxylic acid groups, and it is likely that all deprotonation occurred simultaneously.

### Solvent Extraction

Figure 2 illustrates the distribution ratios of Am­(III), Cm­(III), and Eu­(III) measured as a function of the HNO_3_ concentration. Distribution ratios between 0.01 and 100 exhibit an uncertainty of ±5%, while lower/higher values exhibit larger uncertainties. It has been demonstrated that an increase in the HNO_3_ concentration is indicative of an increase in the distribution ratios of all metal ions. This tendency is expected given that at high nitrate concentrations, TODGA exhibits a stronger complexation. In the case of increasing the concentration of HNO_3_ (up to 1 M), the separation factor (SF_Eu/Am_) of Eu compared to that of Am decreased significantly.

**Figure 2:**
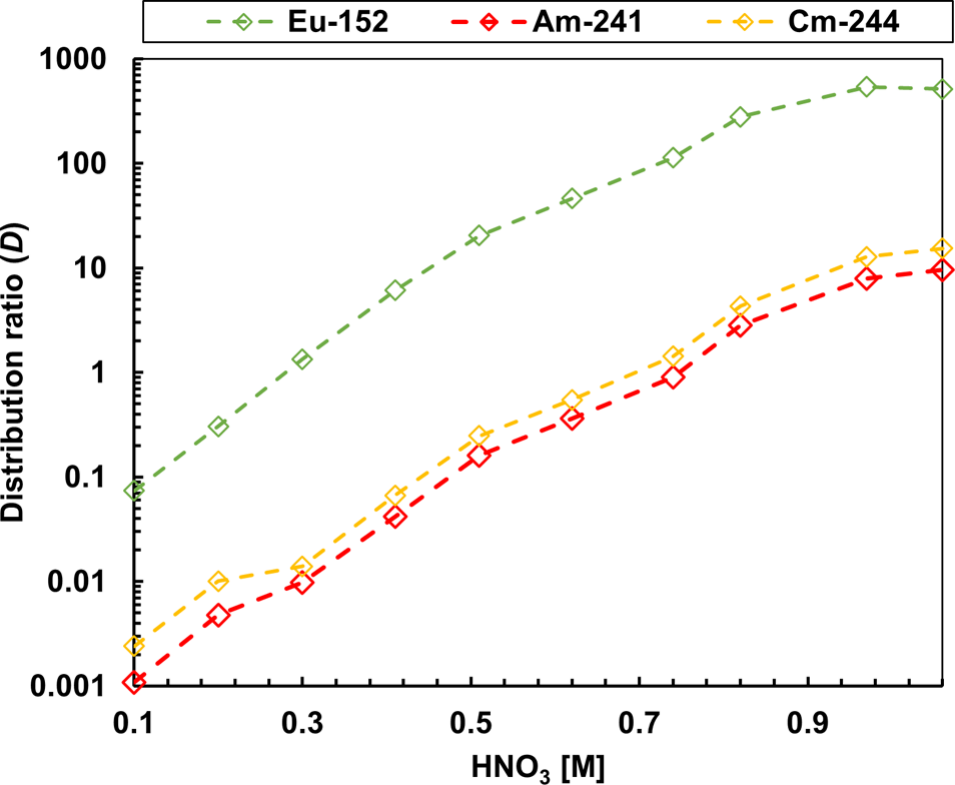
Distribution ratios as a function of the HNO_3_ concentration. Exp. Cond.: Org: 0.2 M TODGA in 5 vol % octanol/TPH. Aq.: 0.01 M BTPOA in HNO_3_ with 10 μM of each Ln­(III) (w/o Pm), spiked with ^241^Am­(III), ^244^Cm­(III), and ^152^Eu­(III) at 22 °C and an O/A = 1.

Based on these data, the best concentrations for the performance of BTPOA and highest separation of An­(III) and Ln­(III) are 0.3–0.7 M HNO_3_.

The next step was to compare the separation factors to determine whether the ligand can be a potential CHON candidate for the *i*-SANEX system as a replacement for SO_3_-Ph-BTP. The separation factors are calculated as the quotient between the distribution ratios of two metals (*q.v.* SI-Methodology). Table 1 presents a comparison among the TODGA systems when used alone and in combination with either SO_3_-Ph-BTP or BTPOA.

**Table 1:** Comparison of the Separation Factors in Different Systems^a^

SF	TODGA	TODGA/BTPOA	TODGA/SO_3_-Ph-BTP^12^
SF_Eu(III)/Am(III)_	∼6	50–120	250–1000
SF_*Cm*(III)/Am(III)_	∼1.3	∼1.5	∼1

a Exp. cond.: separation factors are from 0.1 to 1 M HNO_3_.

The combination of soft and hard donor atoms in BTPOA yields a synergistic effect,^17,51,52^ which improves the resulting SF_Eu(III)/Am(III)_. BTPOA achieves high separation factors between Ln­(III) and An­(III), with a considerable preference for An over Ln, in comparison with TODGA alone. However, the SF_Eu(III)/Am(III)_ reached by SO_3_-Ph-BTP was higher than that obtained with BTPOA. The octa-acid is nevertheless suitable as a replacement for SO_3_-Ph-BTP in the *i*-SANEX system. The SF_*Cm*(III)/Am(III)_ remains constant and unaltered by the concentration of HNO_3_, with the SF corresponding mainly to the influence of TODGA. In this case, BTPOA shows a higher selectivity compared to that of SO_3_-Ph-BTP.

We performed forward and backward extraction tests to observe the rate at which BTPOA forms complexes with the metal ions of interest. The difference between both tests relies on the number of steps involved. In the forward extraction, a single-step reaction, the organic phase contains the extractant, while the aqueous phase contains BTPOA, which has been dissolved in HNO_3_ and mixed with the metal ions and radioactive tracers. In contrast, backward extraction comprises two steps. The first one involves loading the organic phase with the metals, Ln­(III) and An­(III), followed by the second step, which entails the An­(III) being back-extracted into the aqueous phase with BTPOA. Figure 3 shows the distribution ratios of the selected metal ions at different extraction times.

**Figure 3:**
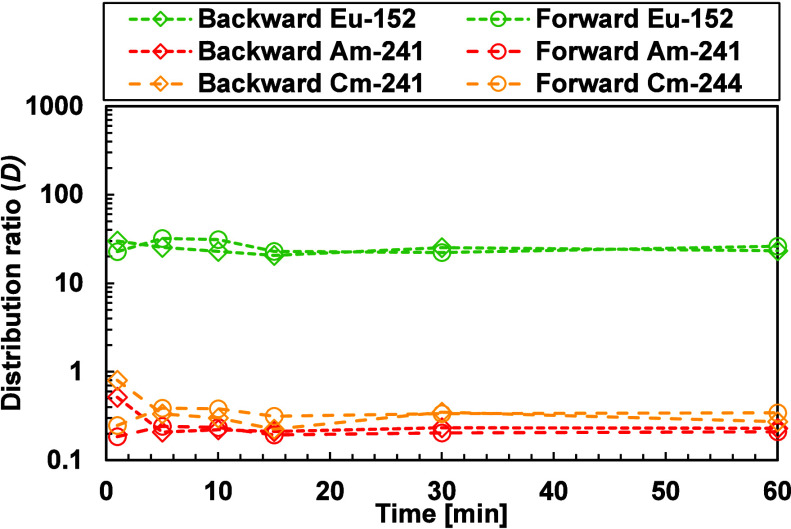
Forward and backward extractions as a function of time. Exp. cond.: Org.: 0.2 M TODGA in 5 vol % octanol/ISANE IP175. Aq.: 0.01 M BTPOA in 0.5 M HNO_3_ with 10 μM Ln­(III) (w/o Pm), spiked with ^241^Am­(III), ^244^Cm­(III), and ^152^Eu­(III) at 22 °C and an O/A = 1.

The results indicated that BTPOA complexes with ^241^Am, ^244^Cm, and ^152^Eu are formed in less than 5 min and remain stable over time, independent of the extraction direction.

The kinetic results for Ln­(III) display different behavior between the light and heavy lanthanides. The light Ln­(III)­s exhibit fast kinetics for both extraction modes, which give consistent kinetic rate coefficient values. For the heavy Ln­(III)­s, there is a visible difference between forward and backward extraction modes, as shown in Figures 4 and 5, respectively. In the backward extraction, equilibrium is reached after 30 min, whereas in the forward extraction, equilibrium is attained after 50–60 min. This difference might be attributed to the size and charge density of the metal ions. Moreover, in the context of forward extraction, a competitive dynamic exists between TODGA and BTPOA. Conversely, in the process of backward extraction, the extraction and back-extraction stages are executed by a single agent.

**Figure 4:**
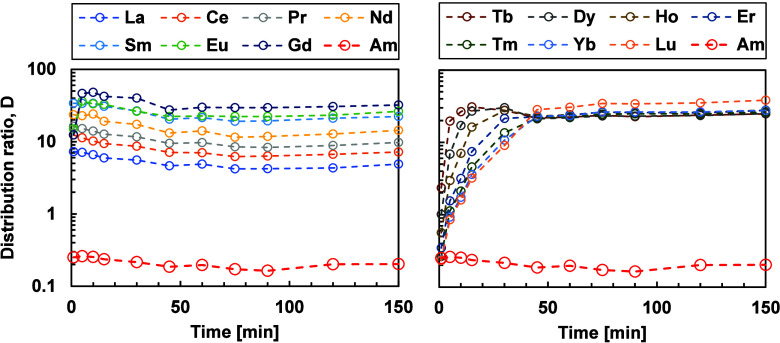
Forward extraction of the lanthanide series compared with Am­(III). (Left: light Ln, right: heavy Ln) as a function of time. Exp. cond.: Org: 0.2 M TODGA in 5 vol % octanol/ISANE IP175. Aq.: 0.01 M BTPOA in 0.5 M HNO_3_ with 10 μM Ln­(III) (w/o Pm), spiked with ^241^Am­(III), ^244^Cm­(III), and ^152^Eu­(III) at 22 °C and an O/A = 1.

**Figure 5:**
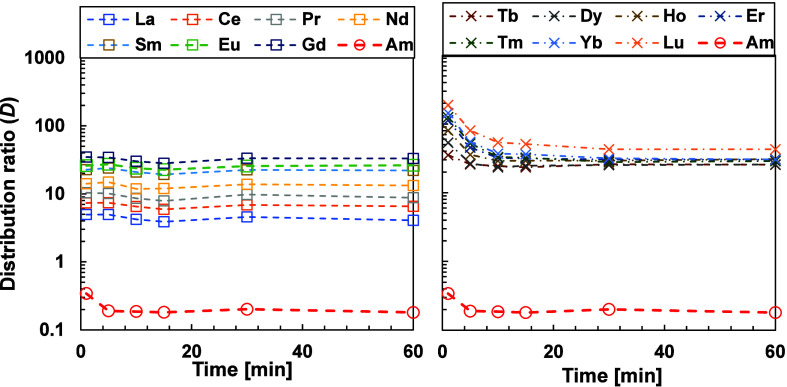
Backward extraction of the lanthanide series compared with that of Am­(III). (Left: light Ln, right: heavy Ln) as a function of time. Exp. cond.: Org.: 0.2 M TODGA in 5 vol % octanol/ISANE IP175. Aq.: 0.01 M BTPOA in 0.5 M HNO_3_ with 10 μM Ln­(III) (w/o Pm), spiked with ^241^Am­(III), ^244^Cm­(III), and ^152^Eu­(III) at 22 °C and an O/A = 1.

As an alternative hypothesis, the salting-in/out effect may be a contributing factor because the salting-out effect has been shown to increase the extraction rate and distribution ratios reflected in the backward extraction with the amount of NO_3_^–^.^53^ The enhancement of extraction results has been demonstrated to increase the density of the aqueous phase. An alternative method that has been observed to yield a similar effect is the increase in the number of extractions, which consequently elevates the recovery rate.^54,55^ In the case of BTPOA, a higher NO_3_^–^ concentration inhibits the extraction of the metal ion into the organic phase.

The complexation of Am­(III), Cm­(III), and Eu­(III) with BTPOA was studied as a function of the ligand concentration, as illustrated in Figure 6. This can be assessed using the slope analysis method, which is a graphical tool whose slope corresponds to the stoichiometric coefficients of the postulated chemical reaction for the ligand of interest. The bistriazinyl family obeys the following reaction (eq 9) for organic ligands:^14^

$$M_{\mathrm{aq}}^{3+}+3{\mathrm{NO}}_{3}^{-}+3L_{\mathrm{org}}⇌M(L{)}_{3}({\mathrm{NO}}_{3}{)}_{3,\mathrm{org}}$$ 9

where M^3+^ corresponds to the metal ion, in our case An­(III) or Ln­(III), L denotes the organic ligand, NO_3_^–^ corresponds to the nitrates present in the extraction, and the product of the reaction is the complex just formed.

**Figure 6:**
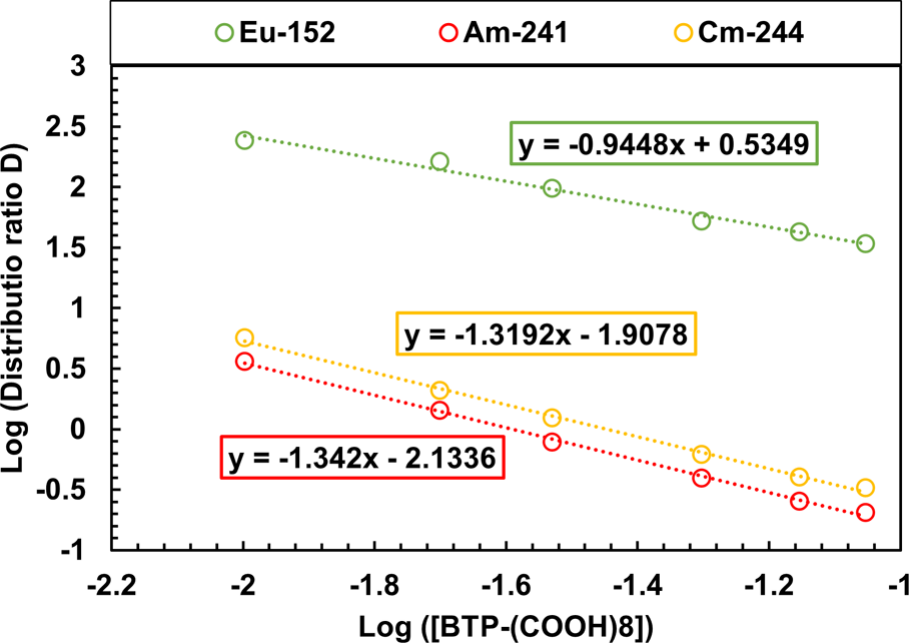
Am­(III), Cm­(III), and Eu­(III) distribution ratios vs ligand concentration. Exp. cond: Org.: 0.2 M TODGA in 5 vol % octanol/TPH; Aq.: BTPOA acid in 0.82 M HNO_3_ with 10 μM Ln­(III) (w/o Pm), spiked with ^241^Am­(III), ^244^Cm­(III), and ^152^Eu­(III) at 22 °C and an O/A = 1.

However, the stoichiometry changes for water-soluble ligands have been postulated by Ruff et al.^56^ for the reaction of water-soluble BTP during the back-extraction process. The first step is loading of the organic phase with the corresponding metals (eq 9). Afterward, the hydrophilic ligand complexes the metals, with the bistriazinyl group selectivity being higher for An­(III) over Ln­(III), the probability of occurrence for eq 10 is higher than that of eq 11:

$$[\mathrm{An}({\mathrm{NO}}_{3}{)}_{3}(\mathrm{TODGA}{)}_{3}{)}_{\mathrm{org}}]+3\mathrm{BTPOA}\overset{K_{\mathrm{ex}}}{↔}[\mathrm{An}(\mathrm{BTPOA}{)}_{3}{]}_{\mathrm{aq}}^{x+}+3{\mathrm{NO}}_{3}^{-}+3{\mathrm{TODGA}}_{\mathrm{org}}$$ 10

$$[\ln({\mathrm{NO}}_{3}{)}_{3}(\mathrm{TODGA}{)}_{3}{)}_{\mathrm{org}}]+3\mathrm{BTPOA}\overset{K_{\mathrm{ex}}}{↔}[\ln(\mathrm{BTPOA}{)}_{3}{]}_{\mathrm{aq}}^{x+}+3{\mathrm{NO}}_{3}^{-}+3{\mathrm{TODGA}}_{\mathrm{org}}$$ 11

where M^3+^ corresponds to the metal ion, in our case An­(III) or Ln­(III), L denotes the organic ligand, and *K*_ex,aq_ is the extraction equilibrium constant. The *K*_ex,aq_ is then defined as:

$$K_{\mathrm{ex},\mathrm{aq}}=\frac{[\mathrm{An}(\mathrm{BTPOA}){]}_{\mathrm{aq}}^{x+}\times [{\mathrm{NO}}_{3}^{-}{]}^{3}\times [{\mathrm{TODGA}}_{\mathrm{org}}{]}^{3}}{[\mathrm{An}({\mathrm{NO}}_{3}{)}_{3}(\mathrm{TODGA}{)}_{3}{)}_{\mathrm{org}}]\times [\mathrm{BTPOA}{]}^{x+}}$$ 12

The double-logarithmic graph of the distribution ratio and ligand concentration gives a linear equation, with the slope corresponding to the stoichiometric coefficients of the ligand. This enables calculation of the metal-to-ligand ratio. The slopes for Am­(III), Cm­(III), and Eu­(III) were 1.34 ± 0.03, 1.32 ± 0.03, and 0.95 ± 0.06, respectively. These slopes differ significantly from an expected value of 3, as literature data suggests 1:3 complexes for BTP-type ligands.^14,56^ Previous studies have reported inconsistent results in slope analysis for hydrophilic complexants with bistriazinyl–TODGA systems with slopes one order less than the real ratio.^14^ However, in this case, the slope analysis resulted in two units less than the expected number.^14,18,56^ Therefore, it was deemed necessary to determine the correct metal/ligand ratio with another technique. Thus, TRLFS measurements were performed to further elucidate this chemistry (see later).

To observe the possible coextraction of An­(III) or Ln­(III) with other transition metals present in UNF and PUREX raffinates, BTPOA was tested over simulated high active raffinate (HAR), which contains light Ln­(III) and other fission products (Ru, Rb, Sr, Zr, Mo, etc., see SI). During the loading of the organic phase, most of the non-Ln­(III) fission products remained in the aqueous phase, and only Sr was extracted together with the light Ln­(III). In the scrubbing step, ^241^Am­(III), ^152^Eu­(III), and ^244^Cm­(III) were added and extracted into the organic phase, while most of the Sr­(II) was back-extracted into the aqueous phase, leaving the loaded TODGA phase with minimal amounts of the non-Ln­(III) fission products. In the stripping step, light Ln­(III) and Y­(III) remained in the organic phase, and Am­(III) and Cm­(III) were selectively back-extracted into the aqueous phase.

Figure 7 presents the distribution ratios of Ln­(III), Am­(III), and Cm­(III) as a function of the HNO_3_ concentration in the stripping step. Metal ion distribution ratios increased with increasing HNO_3_ concentration, comparable to Figure 2. Due to the partial loading of TODGA, generally lower distribution ratios were observed, and La­(III), Ce­(III), and Pr­(III) distribution ratios were <1 at the lowest tested HNO_3_ concentration. Still, good selectivity between An­(III) and all Ln­(III)­s was achieved, and between 0.3 and 0.5 M HNO_3_ with high separation factors (e.g., SF_La/Am_= 16 at 0.5 M), a selective separation of An­(III) seems possible.

**Figure 7:**
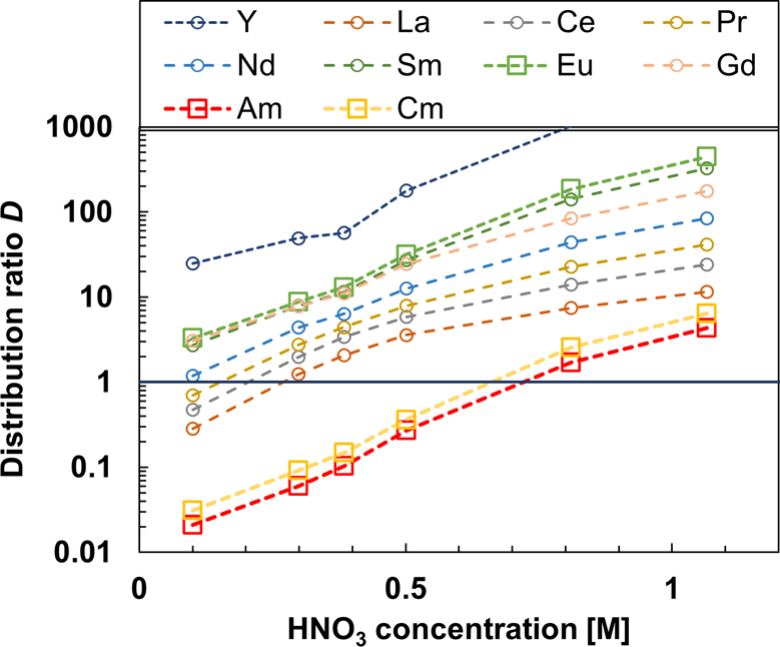
Experimental results of demonstration of the *i*-SANEX system. Exp. cond.: Org.: 0.2 M TODGA in 5 vol % octanol/ISANE IP175. Aq.: step 1:2.96 M HNO_3_ HAR (Ln­(III) and metals, see Table S1), step 2:0.5 M HNO_3_ and spiked with ^241^Am­(III), ^244^Cm­(III), and ^152^Eu­(III). Step 3:0.01 M BTPOA in HNO_3_ at 22 °C and an O/A = 1.

The extraction process was also tested at different temperatures. Figure 8 shows Am­(III), Cm­(III), and Eu­(III) distribution ratios as a function of the temperature. The distribution ratios decreased with increasing temperature, indicating that the system is exothermic, which is also observed in the TODGA system.

**Figure 8:**
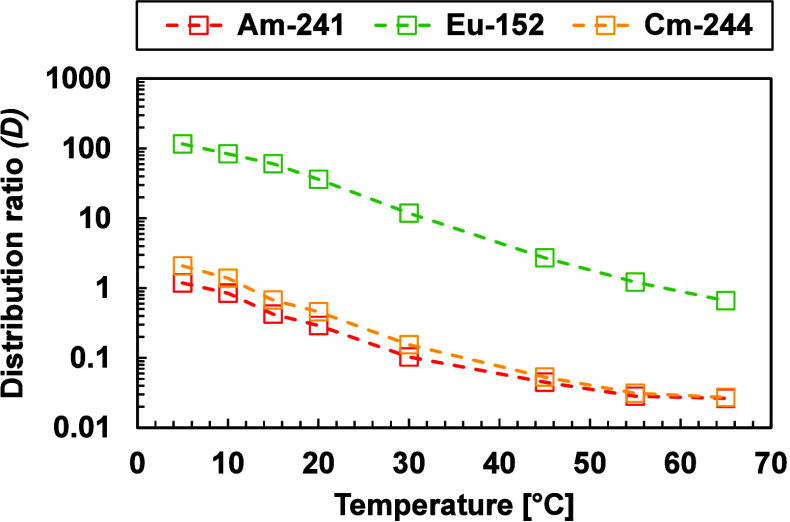
Distribution ratios of Am­(III), Cm­(III), and Eu­(III) at 0.5 M HNO_3_ as a function of the temperature (°C). Exp. cond.: Org.: 0.2 M TODGA in 5 vol % octanol/ISANE IP175. Aq.: 0.01 M BTPOA in 0.5 M HNO_3_ with 10 μM Ln­(III) (w/o Pm), spiked with ^241^Am­(III), ^244^Cm­(III), and ^152^Eu­(III) at an O/A = 1. Time: 30 min.

For solvent extraction thermodynamics studies, the exact stoichiometry and interaction of all ions and molecules involved in the reaction needs to be known, which is difficult to determine with two ligands in different phases. In this case, the TODGA stoichiometry is assumed to be 1:3,^57^ with three additional NO_3_^–^ involved in the complexation.^15^ For BTPOA, a 1:3 species is currently assumed (see TRLFS section), but the amount of NO_3_^–^ or water molecules is not confirmed. Therefore, further calculations, such as complexation data at different temperatures with each ligand and in combination with the available data, cannot be done.

As many of the hydrometallurgical processes for UNF management are carried out in HNO_3_, it is crucial to obtain long-term knowledge of the stability of the ligand in acidic media. Hence, BTPOA was dissolved in different concentrations of HNO_3_, and the solution was used in extraction experiments over a period of nine months.

Figure 9 shows the distribution ratios of ^241^Am­(III), ^244^Cm­(III), and ^152^Eu­(III), which present minimal changes after the first week. Six months later, the distribution ratios remained almost the same. The slight variations might be attributed to other factors, not particularly to the degradation of the ligand. After nine months, the distribution ratios started to increase slightly, showing the slow degradation of BTPOA.

**Figure 9:**
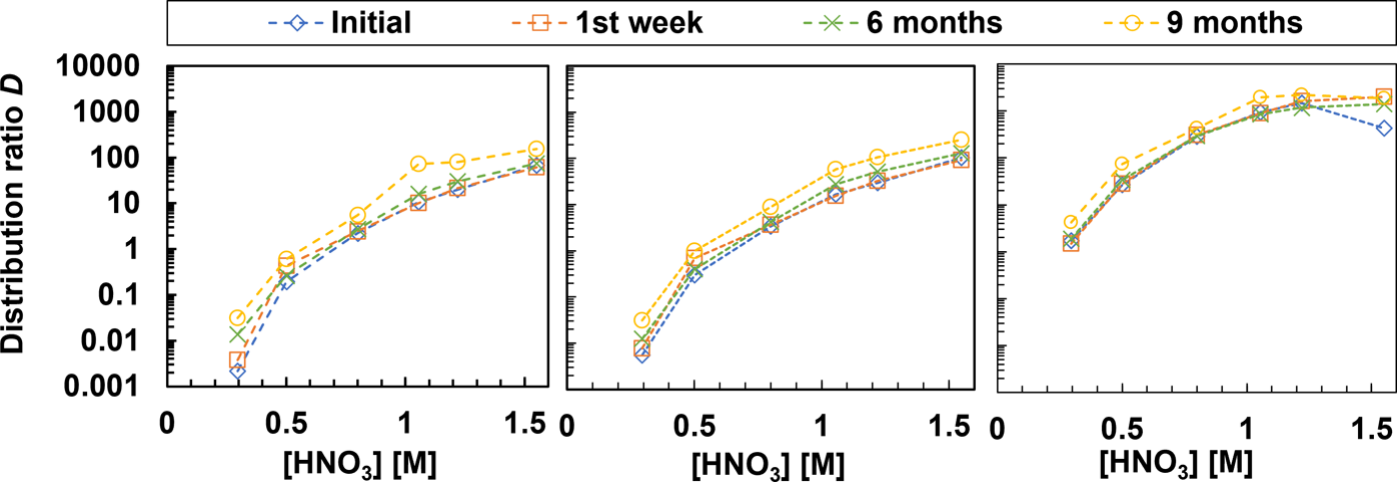
Hydrolysis experiment as a function of HNO_3_ concentration over a 9-month period for ^241^Am­(III) (left), ^244^Cm­(III) (middle), and ^152^Eu­(III) (right). Exp. cond.: Org.: 0.2 M TODGA in 5 vol % octanol/ISANE IP175. Aq.: 0.01 M BTPOA in HNO_3_ with 10 μM Ln­(III) (w/o Pm) at 22 °C and an O/A = 1.

The Ln­(III) showed similar results at low HNO_3_ concentrations, where the distribution ratios remain unchanged with time. However, as the concentration of HNO_3_ increased, the values changed.

In Figure 10, we can observe the influence of HNO_3_ concentration and the difference between light and heavy Ln­(III)­s, exhibiting, on average, a factor of 40 variation in their distribution ratios. The light Ln­(III)­s follow the expected trend, where the distribution ratios increased with time, whereas for heavy Ln­(III)­s, the distribution ratios decreased over time as the concentration of HNO_3_ increased. This outcome needs to be further investigated. Overall, BTPOA is stable in 0.1–0.8 M HNO_3_ solution for at least six months.

**Figure 10:**
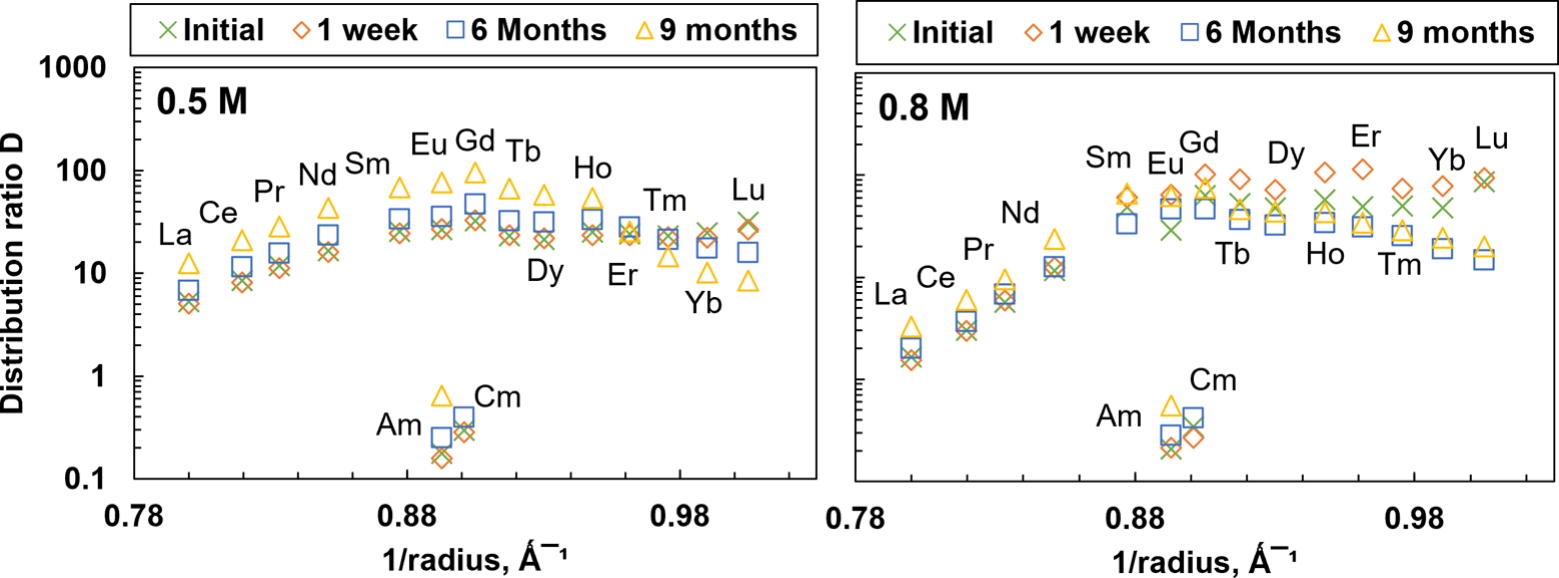
Hydrolysis experiment as a function of 1/radius over a 9-month period for Ln­(III) at 0.5 and 0.8 M HNO_3_. Exp. cond.: Org.: 0.2 M TODGA in 5 vol % octanol/ISANE IP175. Aq.: 0.01 M BTPOA in HNO_3_ with 10 μM Ln­(III) (w/o Pm) at 22 °C and an O/A = 1.

### Speciation Studies Using TRLFS

In the same way that solvent extraction is fundamental to the characterization and assessment of BTPOA, TRLFS can be used to evaluate and complement the complexation data. The complexation of Cm­(III) and Eu­(III) was studied in two acid systems (HClO_4_ and HNO_3_) with a stock solution of 10 μM for Cm­(III) and Eu­(III). HClO_4_ comprises a nonbinding anion, and only water and ligand molecules are expected to bind to the metal ion throughout the titration. In contrast, NO_3_^–^ is a stronger ligand than ClO_4_^–^ and can form weak complexes with metal ions.

Figure 11 shows the progression of the Cm­(III) fluorescence spectra from the ^6^D′_7/2_ to →^8^S′_7/2_ transition with increasing ligand concentration. The Cm­(III) aquo ion shows a single emission band at 593.8 nm.^58^ With increasing ligand concentration, three emission bands with maxima at 600.1, 609.7, and 617.5 nm occur, corresponding to metal/ligand ratios of 1:1, 1:2, and 1:3, respectively. The bathochromic shifts (6, 16, and 24 nm) indicate a metal ion complexation by three nitrogen atoms of the ligands, and the addition of one ligand molecule per complexation step.^17^

**Figure 11:**
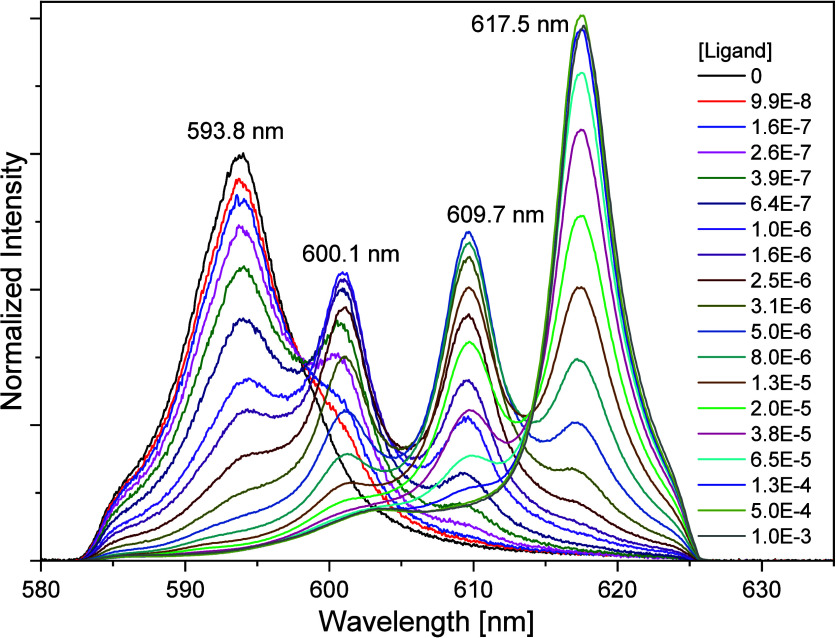
Normalized fluorescence spectra in 1 mM HClO_4_ for the complexation of Cm­(III) with BTPOA as a function of BTPOA concentration (0–1 mM).

The Cm­(III) species distribution is derived by peak deconvolution of the fluorescence spectra using the single-component spectra given in Figure S7. To determine species concentrations, the fluorescence intensity factors (FI factors) of the different species must be considered. The FI factor of a complex species (FI_i_) is calculated from the intensity ratio of species *I*_i_ and a reference species *I*_ref_ (eq 13). The solvent complex represents the reference species, whose FI factor FI_ref_ = 1:

$${\mathrm{FI}}_{i}=\frac{I_{i}}{I_{\mathrm{ref}}}$$ 13

The relative FI factor for the 1:3 complex is 180, while for the other two species it is 1. Figure 12 shows the species distribution as a function of the noncomplexed BTPOA concentration.

**Figure 12:**
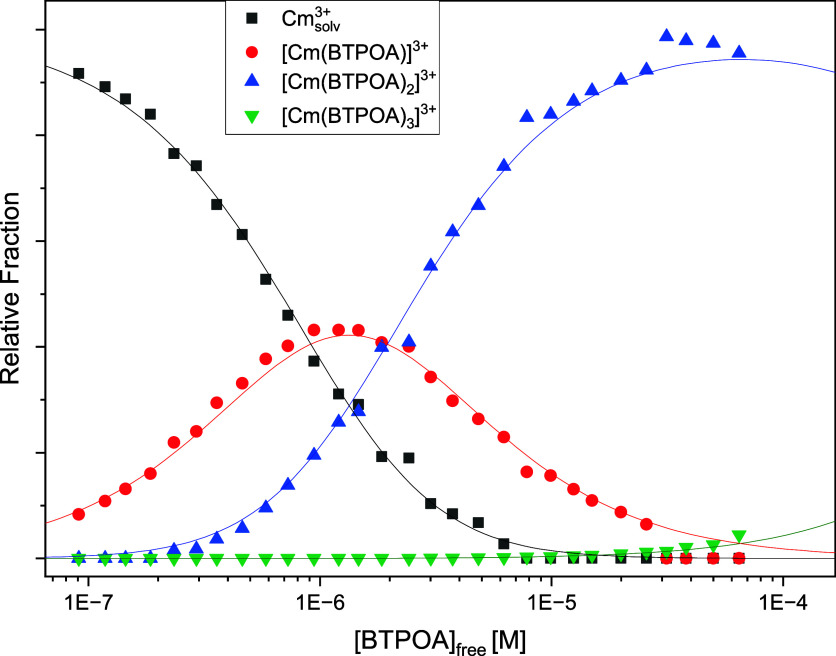
Speciation of Cm­(III) as a function of noncomplexed BTPOA concentration in 1 mM HClO_4_ with its corresponding trend lines.

The noncomplexed ligand concentration was calculated according to eq 14:

$$\left[L{]}_{\mathrm{free}}=0.5\times ((4\times [L{]}_{0}\times K_{a}+[H^{+}{]}_{0}^{2}+2\times [H^{+}{]}_{0}\times K_{a}+K_{a}^{2}{)}^{0.5}-[H^{+}{]}_{0}-K_{a})-([\mathrm{Cm}(\mathrm{III})]\times (\chi_{1:1}+2\times \chi_{1:2}+3\times \chi_{1:3})\right)$$ 14

The complexation of BTPOA is described by eq 15 and the stoichiometry of the complex species can be determined by slope analyses (see Methods section) using the linear correlation between the logarithm of the noncomplexed ligand concentration and the concentration ratio between the species formed (eq 17). The slope analyses for the stepwise formation of the 1:1, 1:2, and 1:3 complex yielding slopes of 1 are shown in Figure S8. The conditional stability constants were calculated by the law of mass action (eqs 16 and 17). For the 1:1 complex logβ′_1_ = 6.06 ± 0.05, for the 1:2 complex logβ′_2_ = 11.75 ± 0.11, and for the final 1:3 complexation step, logβ′_3_ = 16.6 ± 0.1 was determined:

$$M[(\mathrm{solv}{)}_{9}]+nL⇌{\mathrm{ML}}_{n}(n=1,2,3)$$ 15

$$K_{n}=\frac{\left[{\mathrm{ML}}_{n}\right]}{[M(\mathrm{solv}{)}_{9}]\times [L{]}^{n}}$$ 16

$$\log\left(\frac{\left[{\mathrm{ML}}_{n}\right]}{\left[M(\mathrm{solv}{)}_{9}\right]}\right)=\log K+n\log[L]$$ 17

In comparison with SO_3_-Ph-BTP,^59^ the conditional stability constants of BTPOA complexes are larger, implying that it is a stronger binding ligand (Table 2). However, its extraction performance is worse under comparable chemical conditions, possibly due to the high number of carboxylic acid groups, as the deprotonated carboxylic acid groups could also complex the metal ions in addition to the nitrogen atoms from the pyridine and triazine rings, which hinders the extraction process, since the metal is not located in the right coordination site, giving place to faster dissociation or lowering the coordination constants.

**Table 2:** Comparison of Conditional Stability Constants of SO_3_-Ph-BTP and BTPOA in HClO_4_

stability constant	SO_3_-Ph-BTP^59^ ^a^	BTPOA
**log** β′ _**1**_	5.4 ± 0.1	6.0 ± 0.2
**log** β′_**2**_	9.3 ± 0.2	11.8 ± 0.2
**log** β′ _**3**_	12.2 ± 0.3	14.4 ± 0.2

a SO_3_-Ph-BTP exp. cond. [Cm­(ClO_4_)_3_]^3+^ 0.12 μM, pH 3.0 with 1 mM HClO_4_. Equilibrium time: 15 min.

Figure 13 shows the emission spectra of Cm­(III) with BTPOA in the presence of 0.5 M HNO_3_. The emission band at 594.2 nm is assigned to the Cm­(III) aquo species ([Cm­(H_2_O)_9_]^3+^) and the emission band at 597.2 nm to the ([Cm­(H_2_O)_9_NO_3_]^2+^) species, in agreement with Ruff et al.^56^ With increasing BTPOA concentration, two emission bands at 609.5 and 617.8 nm occur. Evaluation of the emission band at 609.5 nm shows that two different 1:2 complexes are formed (Figure S10). The emission band at 617.8 nm corresponds to the 1:3 complex.

**Figure 13:**
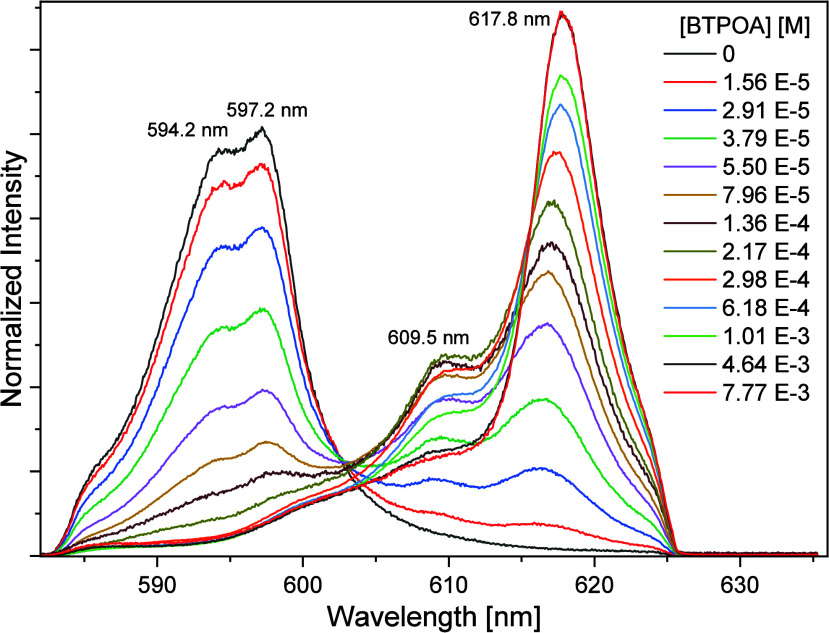
Normalized fluorescence emission spectra in 0.5 M HNO_3_ for the complexation of Cm­(III) with BTPOA as a function of the BTPOA concentration (0–7.77 mM).

A plausible explanation for the existence of two 1:2 complexes displaying different spectroscopic shifts is the replacement of a water molecule in the inner coordination sphere by NO_3_^–^, as the difference in wavelength is too small to be assigned to a different complexation mode (O vs. N).

Since the ligand presents a high FI factor of (116) for the 1:3 species, this FI factor must be considered to determine species concentrations. The species distribution, including the two different 1:2 complex species, is shown in Figure 14.

**Figure 14:**
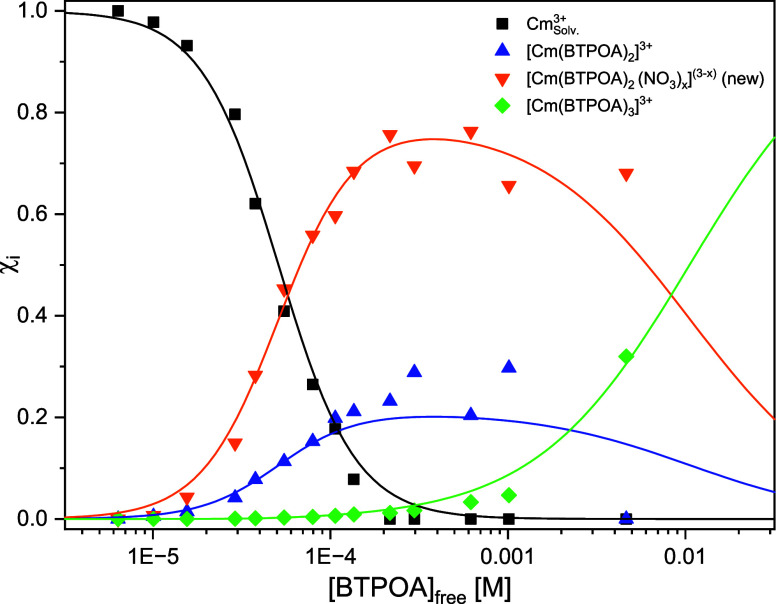
Speciation of Cm­(III) as a function of the concentration of BTPOA in 0.5 M HNO_3_ with its corresponding trend lines.

Table 3 shows a comparison of the conditional stability constants of the Cm 1:3 complexes with BTPOA and SO_3_-Ph-BTP in the HClO_4_ and HNO_3_ systems. In the presence of NO_3_^–^, the stability constant of the 1:3 complex decreases by several orders of magnitude. This is in line with the stronger complexation properties of NO_3_^–^ compared to ClO_4_^–^. However, in the NO_3_^–^ medium, the stability constants of the 1:3 complexes of Cm­(III) with BTPOA and SO_3_-Ph-BTP are comparable.

**Table 3:** Comparison of the Conditional Stability Constants of Cm­(III) of 1:3 Complexes with BTPOA and SO_3_-Ph-BTP^59^ in HClO_4_ and HNO_3_

	0.5 M HNO_3_		1 mM HClO_4_	
log β′_*n*_	BTPOA	SO_3_-Ph-BTP	BTPOA	SO_3_-Ph-BTP^59^
**3**	10.6 ± 0.4	10.6	14.4 ± 0.4	12.2

Additionally, the organic and aqueous phases were measured in TRLFS. These were produced from the extraction using the same conditions as for the solvent extraction experiments. This was done in order to identify which species are formed during extraction.

Figure 15 shows the emission spectrum of Cm­(III) in the aqueous phase after extraction and the [Cm­(BTPOA)_3_]^x+^ complex in HNO_3_ (left)_._ Both spectra are identical, which confirms the formation of 1:3 Cm­(III)-BTPOA under extraction conditions. In the organic phase (right), the spectrum after extraction agrees with the one of the 1:3 Cm­(III) complexes with TODGA in ethanol.^61^ The small deviation in the peak position is caused by the different organic solvents used (TPH and IP175). Thus, it was possible to identify the extraction-relevant Cm­(III) complex species by a comparison with the results of monophasic speciation studies.

**Figure 15:**
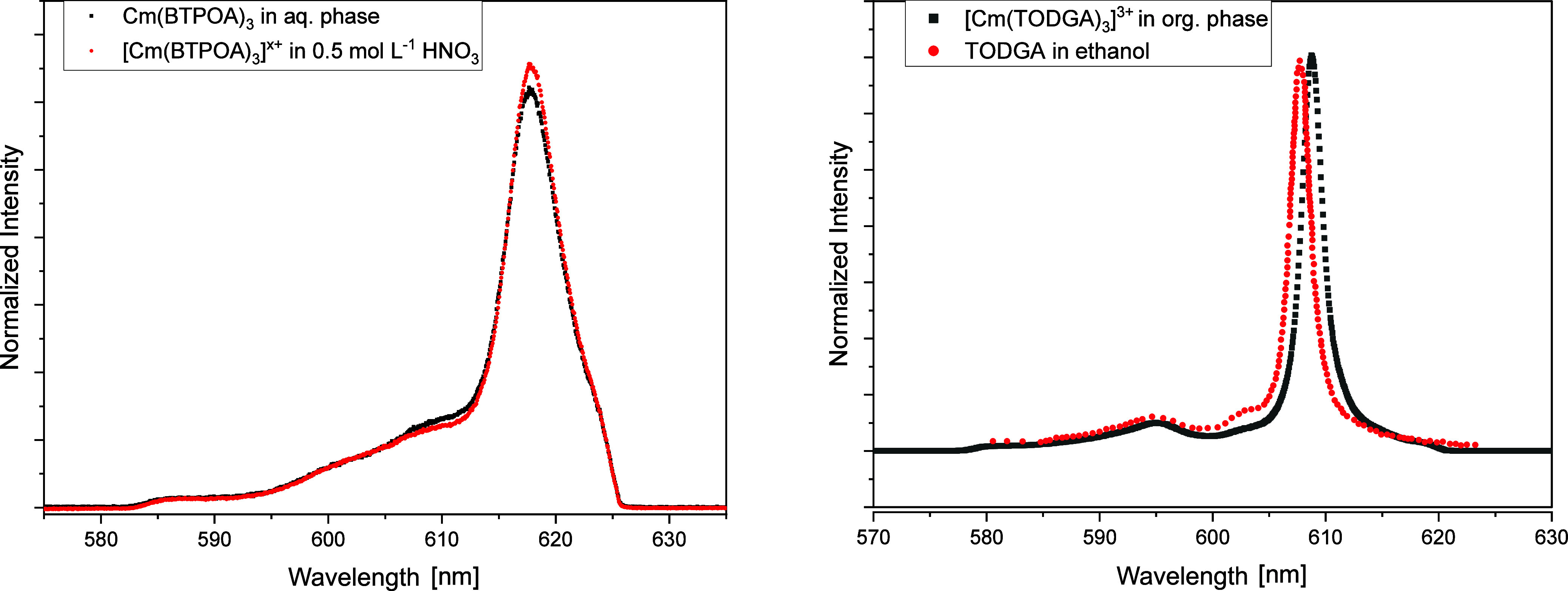
Left: Normalized Cm­(III) spectrum in the aqueous phase after extraction and emission spectrum of [Cm­(BTPOA)_3_]^x+^. Right: Normalized Cm­(III) spectrum in the organic phase after extraction and spectrum of the 1:3 TODGA complex in ethanol.^60^ Exp. cond: organic phase: 0.2 M TODGA in 5 vol % octanol/TPH. Aqueous phase: 10 mM BTPOA spiked with 10 μM Cm­(III).

To study the differences in the complexation properties of BTPOA toward An­(III) and Ln­(III), further speciation studies were performed with Eu­(III). Due to the very high FI factors of the complexed species, quantitative speciation studies of Eu­(III) with BTPOA in HClO_4_ were not possible. However, the FIs were found to be lower in HNO_3_, allowing the titration to be conducted over a wider range of ligand concentrations and the deconvolution of the emission spectra. The normalized emission spectra of Eu­(III) with BTPOA resulting from the ^5^D_0_ → ^7^F_1_ and ^5^D_0_ → ^7^F_2_ transitions are shown in Figure 16.

**Figure 16:**
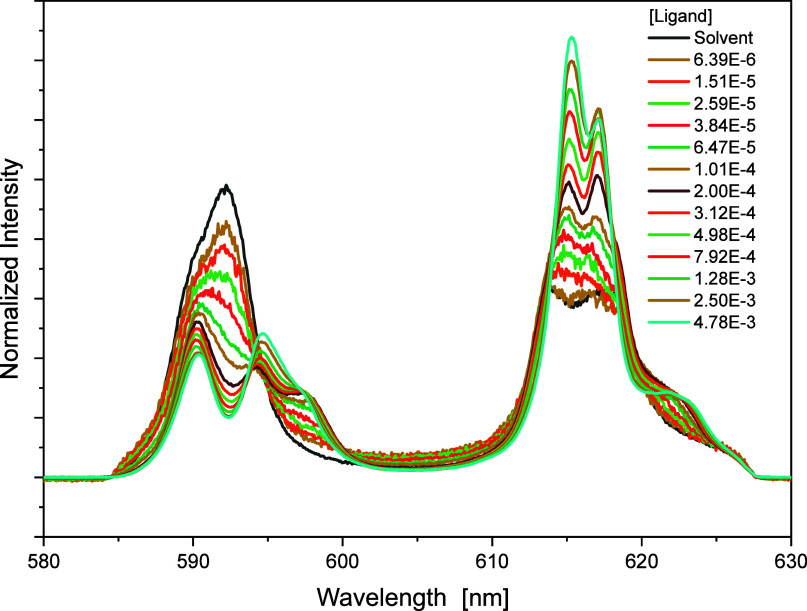
Normalized emission spectra for the complexation of Eu­(III) (^7^F_1_ and ^7^F_2_ bands) with BTPOA in 0.5 M HNO_3_ as a function of the BTPOA concentration.

In the absence of BTPOA, the solvent spectrum of Eu­(III) is characterized by two emission bands at 592 nm (^7^F_1_ band) and 612.2 nm/615.6 nm (^7^F_2_ band). Upon the addition of the ligand, the relative intensity of the ^7^F_1_ band decreases, while two new peaks evolved at 590.45 and 594.51 nm. Regarding the ^7^F_2_ band, two sharp peaks at 615.73 and 617.27 nm are observed with progressive complexation. Deconvolution of the emission spectra showed the formation of three different complex species: [Eu­(BTPOA)_*n*_]^x+^ (*n* = 1, 2, or 3). Again, the FI factor (180) of the 1:3 complex is much higher than those for the other two species, which must be considered when calculating the species concentrations. The species distribution is shown in Figure 17.

**Figure 17:**
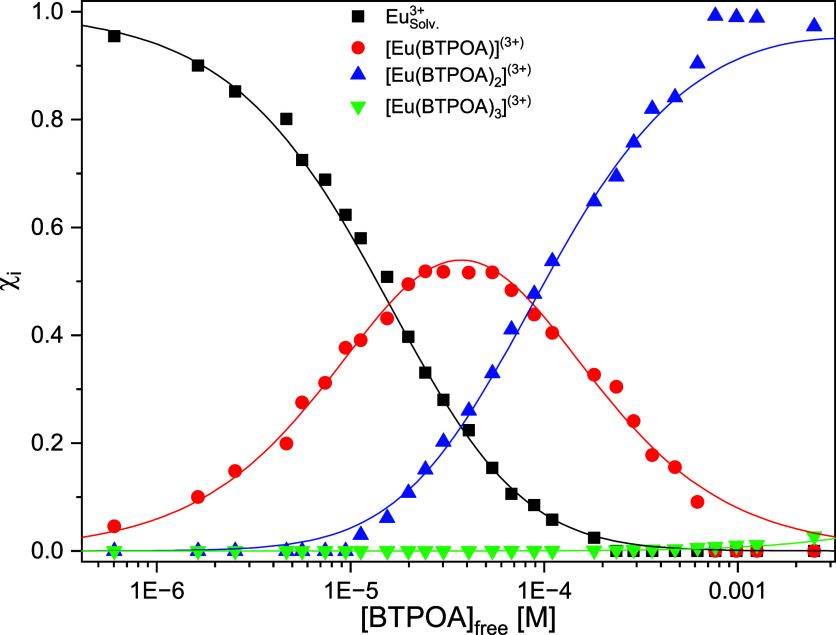
Speciation of Eu­(III) as a function of the concentration of noncomplexed BTPOA in 0.5 M HNO_3_ with its corresponding trend lines.

The stoichiometry of the different Eu­(III) BTPOA complexes is confirmed by slope analyses according to a stepwise complexation model. Plotting the logarithm of ([Eu­(BTPOA)_*n*_]^x+^/Eu­(BTPOA)_*n*−1_]^x+^) as a function of the logarithm of the noncomplexed BTPOA concentration (see Figure S11) results in slopes of 1 for each complex species (*n* = 1, 2, or 3).

Based on the resolved speciation, the corresponding conditional stability constants were calculated: logβ_1_ = 4.9 ± 0.2, logβ_2_ = 8.9 ± 0.3, and logβ_3_ = 9.8 ± 0.4. The stability constants of the extraction-relevant 1:3 complexes for Cm­(III) and Eu­(III) in HNO_3_ differ by 0.8 orders of magnitude, confirming a weaker binding of Ln­(III) compared to that of An­(III). This trend is consistent with and corroborated by the solvent extraction experiments.

### Radiation-Induced BTPOA Reaction Kinetics

Another key property in the evaluation of potential UNF extractants is their behavior in ionizing radiation fields, as this dictates their viability under the envisioned process conditions. In this regard, radical reaction kinetics provide valuable mechanistic insights that can be leveraged to support computational predictions on a ligand’s radiolytic longevity. The reaction kinetics for this study were measured using the Notre Dame Radiation Research Laboratory (NDRL) pico-to-nanosecond electron-pulsed linear accelerator (LINAC) and transient absorption detection system.^62,63^ The results are summarized in Table 4, along with previously derived values for SO_3_-Ph-BTP.

**Table 4:** Summary of Measured Second-Order Rate Coefficients for the Reaction of BTPOA with Aqueous Phase Transient Radiolysis Products in Comparison to Values for Its Sulfonated Analogue, SO_3_-Ph-BTP

	second-order rate coefficient (*k*, M^–1^ s^–1^)			
ligand	e_aq_^–^ (× 10^10^)	H^•^ (× 10^9^)	^•^OH (× 10^9^)	NO_3_^•^ (× 10^7^)
SO_3_-Ph-BTP^39^	1.51 ± 0.01	3.07 ± 0.11	2.38 ± 0.14	3.72 ± 0.13
BTPOA	1.60 ± 0.02	2.17 ± 0.03	6.95 ± 0.06	0.37 ± 0.02

Concerning the chemically reducing radiolysis products (e_aq_^–^ and H^•^), the substitution of sulfophenyl groups (SO_3_-Ph-BTP) for octa acid functionalities (BTPOA) was found to have a negligible impact on the ligand’s reactivity (Table 4, SI Figures S12 and S13, respectively). This observation indicates that reduction does not take place at the hydrophilic functional groups (R–SO_4_H nor R–CO_2_H), but instead with the BTP core structure. Given similar measurements for the reaction of the e_aq_^–^ with aromatic pyridine (*k* = 3.7 × 10^9^ M^–1^ s^–1^)^64^ and triazine (*k* = 5.2 × 10^9^ M^–1^ s^–1^)^65^ containing molecules, we can assume that the electron reaction occurs at both these moieties within BTPOA. For the H^•^ reaction, the much slower rate coefficients for its reaction with pyridine (*k* = 2.2 × 10^8^ M^–1^ s^–1^)^66^ suggests that the majority of H^•^-reactivity occurs with the triazine moiety in this molecule (no specific triazine literature rate coefficient could be found).

Although these new reducing radical reaction rate coefficients were measured under optimal solution conditions, such as at neutral pH for e_aq_^–^, they are still important for the development of comprehensive predictive models for the radiation-induced behavior of BTPOA. Based on these kinetic data, it can readily be seen that under envisioned process conditions—aerated, aqueous HNO_3_ solution—these reducing transient species are rapidly scavenged by NO_3_^–^ (eqs 4 and 5), acidic protons (H_aq_^+^, eq 18), and dissolved O_2_ (eqs 19 and 20):^40^


$$\begin{array}{cc} {e_{\mathrm{aq}}}^{-}+{H_{\mathrm{aq}}}^{+}\to H^{•} & k_{17}=2.3\times 10^{10}\;M^{-1}s^{-1} \end{array}$$ 18

$$\begin{array}{cc} {e_{\mathrm{aq}}}^{-}+O_{2}\to {O_{2}}^{•-} & k_{18}=1.9\times 10^{10}\;M^{-1}s^{-1} \end{array}$$ 19

$$\begin{array}{cc} H^{•}+O_{2}\to {{\mathrm{HO}}_{2}}^{•} & k_{19}=1.2\times 10^{10}\;M^{-1}s^{-1} \end{array}$$ 20

and thus, likely unavailable for reaction with BTPOA. Consequently, the radiolytic behavior of BTPOA is expected to be driven by the oxidizing products of aqueous HNO_3_ radiolysis, specifically ^•^OH and NO_3_^•^.

Under dilute HNO_3_ conditions, there is only a small amount of undissociated HNO_3_ (eq 3), and thus, ^•^OH will be competitively available for reaction with BTPOA. This reaction was found to give a strongly absorbing transient species, as shown in Figure 18A.

**Figure 18:**
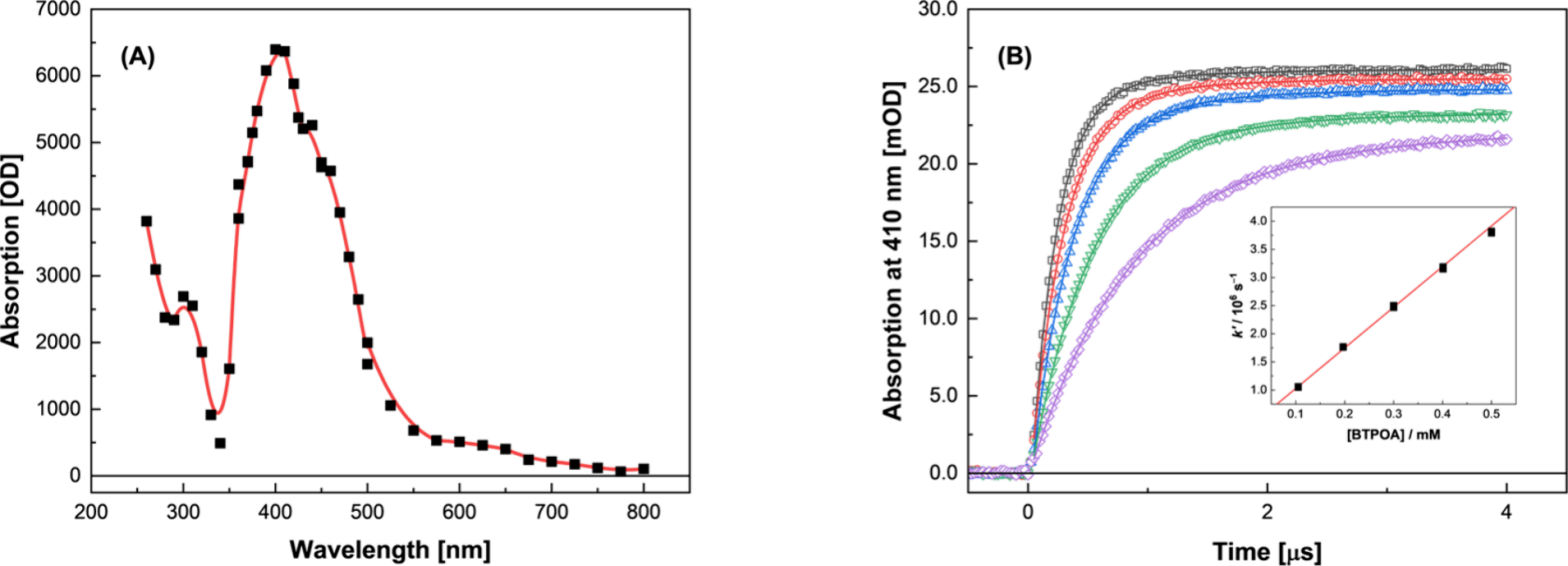
(A) Transient absorption spectra arising from the electron pulse irradiation of 500 μM BTPOA in N_2_O-saturated 10 mM phosphate buffer solution at pH 7.0 ± 0.1 and 24.6 °C. (B) Corresponding pseudo-first-order transient growth kinetics observed at 410 nm for 105 (purple), 197 (green), 300 (blue), 401 (red), and 500 (gray) μM BTPOA. *Inset:* Second-order rate coefficient determination using the fitted pseudo-first-order component values: (1.09 ± 0.01) × 10^6^, (1.85 ± 0.01) × 10^6^, (2.63 ± 0.01) × 10^6^, (3.37 ± 0.02) × 10^6^, and (4.20 ± 0.03) × 10^6^ s^–1^, respectively. The solid line is a weighted linear fit to transformed data, corresponding to *k*(BTPOA + ^•^OH) = (6.95 ± 0.06) × 10^9^ M^–1^ s^–1^, *R* ^2^ = 0.998.

By monitoring the growth rate of this species’ absorption at 410 nm (Figure 18B) and fitting these kinetics to an exponential growth equation:

$$k={\mathrm{Abs}}^{o}(1-e^{-k\prime t})+B$$ 21

where Abs^*o*^ is the limiting transient absorbance, *k*′ is the pseudo-first-order rate coefficient for ^•^OH + BTPOA, *t* is time, and *B* is a baseline adjustment parameter, a plot of *k′* vs [BTPOA] then gives a straight line (see Figure 18B, *Inset*) whose slope corresponds to a second-order rate coefficient of *k* = (6.95 ± 0.06) × 10^9^ M^–1^ s^–1^. This value is more than twice as fast as that previously measured for the analogous SO_3_-Ph-BTP molecule (Table 4), indicating that there is chemical reactivity between the ^•^OH and the hydrophilic BTP functional groups, with the reaction at the carboxyl groups being more energetically favorable than at the sulfonate groups. The ^•^OH typically removes a beta-hydrogen atom from carboxylic acid molecules;^40^ however, BTPOA does not possess beta-hydrogen atoms adjacent to its hydrophilic functional groups. As such, we anticipate that the ^•^OH will add to the aromatic rings adjacent to BTPOA’s carboxyl groups, thereby forming the observed transient adduct, cyclohexadienyl-type, species. This is in keeping with the chemical interactions between the ^•^OH and other aromatic compounds.^40^ The greater susceptibility of BTPOA vs SO_3_-Ph-BTP to the reaction with ^•^OH may be attributed to a combination of: (*i*) differences in the electron richness of the adjacent aromatic rings, due to the lower electron withdrawing capacity of R-COOH (Hammett constant, *s* = −0.37) vs R-SO_3_H (*s* = −0.51);^67^ and (*ii*) changes in steric hindrance, with the sulfonate groups being larger, thereby somewhat shielding the aromatic rings.

Under more concentrated HNO_3_ conditions, the ^•^OH is completely replaced by NO_3_^•^ (eq 6), which has also been shown to exhibit significant reactivity toward reprocessing ligands. As with ^•^OH, the reaction between BTPOA and NO_3_^•^ afforded an observable transient species, the absorption spectrum for which is given in Figure 19A. The spectrum of this species closely resembles that from the ^•^OH reaction (Figure 18A), suggesting a perturbation similar to that of the triazine/pyridine rings.

**Figure 19:**
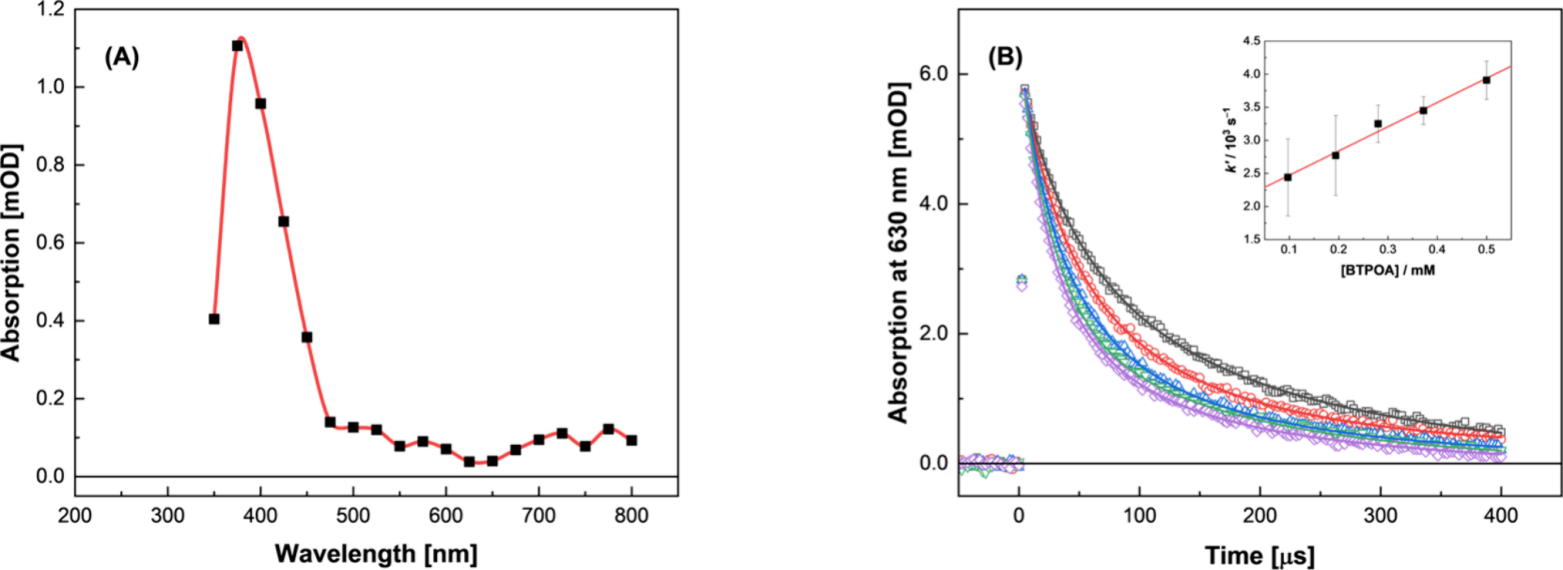
(A) Transient absorption spectra arising from the electron pulse irradiation of 500 μM BTPOA in a 6.0 M HNO_3_ solution at 24.5 °C. (B) Corresponding double first-order transient decay kinetics observed at 630 nm for 97.1 (gray), 194 (red), 280 (blue), 372 (green), and 500 (purple) μM BTPOA. *Inset:* Second-order rate coefficient determination using the fitted pseudo first-order component values (1.53 ± 0.07) × 10^4^, (1.97 ± 0.05) × 10^4^, (2.26 ± 0.04) × 10^4^, (2.77 ± 0.07) × 10^4^, and (3.14 ± 0.08) × 10^4^ s^–1^, respectively. The solid line is a weighted linear fit to transformed data, corresponding to *k*(BTPOA + NO_3_^•^) = (3.70 ± 0.20) × 10^6^ M^–1^ s^–1^, *R* ^2^ = 0.987.

Concerning the reaction kinetics for this process, the decay of NO_3_^•^ was directly monitored at 630 nm as a function of the concentration of BTPOA. The measured decays, shown in Figure 19B, were fit using two first-order decay functions:

$$\mathrm{Abs}={A_{1}}^{*}\exp\left(-k_{\mathrm{BTPOA}}^{’}t\right)+{A_{2}}^{*}\exp\left(-k^{’}t\right)+B$$ 22

where Abs is the transient absorption intensity of NO_3_^•^, *k′*_BTPOA_ and *k′* are the pseudo-first-order rate coefficients for the NO_3_^•^ reaction with BTPOA and other aqueous HNO_3_ radiolysis product species, respectively:^40^


$$\begin{array}{cc} {{\mathrm{NO}}_{3}}^{•}+{\mathrm{OH}}^{•}\to {{\mathrm{NO}}_{2}}^{•}+{{\mathrm{HO}}_{2}}^{•} & k=1.0\times 10^{10}\;M^{-1}s^{-1} \end{array}$$ 23

$$\begin{array}{cc} {{\mathrm{NO}}_{3}}^{•}+H_{2}O\to {\mathrm{HNO}}_{3}+{\mathrm{OH}}^{•} & k=300\;M^{-1}s^{-1} \end{array}$$ 24

$$\begin{array}{cc} {{\mathrm{NO}}_{3}}^{•}+{{\mathrm{NO}}_{2}}^{-}\to {{\mathrm{NO}}_{3}}^{-}+{{\mathrm{NO}}_{2}}^{•} & k=4.4\times 10^{9}\;M^{-1}s^{-1} \end{array}$$ 25

$$\begin{array}{cc} {{\mathrm{NO}}_{3}}^{•}+{{\mathrm{NO}}_{2}}^{•}\to N_{2}O_{5} & k=1.1\times 10^{9}\;M^{-1}s^{-1} \end{array}$$ 26

$$\begin{array}{cc} {{\mathrm{NO}}_{3}}^{•}+{\mathrm{HNO}}_{2}\to {\mathrm{HNO}}_{3}+{{\mathrm{NO}}_{2}}^{•} & k=2.0\times 10^{8}\;M^{-1}s^{-1} \end{array}$$ 27

A_1_ and A_2_ are the corresponding fractions of NO_3_^•^ reacting by each pathway, and B accommodates for any limiting product absorption, i.e., a baseline shift. The *k′*_BTPOA_-fitted values were then plotted against concentration (Figure 19B, *Inset*) to give the second-order rate coefficient, as shown in Table 4. Interestingly, the reaction of BTPOA with NO_3_^•^ is an order of magnitude slower than that of the corresponding SO_3_-Ph-BTP reaction. This difference is contrary to our previous ^•^OH findings.

Although gamma-dose accumulation studies were attempted—using the NDRL’s Nordion Gammacell 220 and a Shepherd 109–68 cobalt-60 irradiators—characterization of the resulting samples was complicated by the BTPOA molecule’s tendency to fragment during LC analysis. In lieu of such experimental results, the above measured reaction kinetics can be used to make some assumptions about the radiolytic longevity of BTPOA under envisioned process conditions. This is especially true given that previous calculations found that the gamma radiolysis of SO_3_-Ph-BTP was predominantly driven (∼90%) by its reaction with ^•^OH in aqueous solutions.^40^ In the case of BTPOA, we anticipate this molecule to exhibit greater longevity in a gamma radiation field as compared to SO_3_-Ph-BTP under the optimized process conditions outlined earlier: 0.3–0.7 M HNO_3_. This is because NO_3_^•^ will be the predominant oxidant, not ^•^OH, which reacts an order of magnitude slower with BTPOA compared to SO_3_-Ph-BTP (Table 4). This is a fortuitous finding for advancing flowsheet development for BTPOA-based separations technology.

## Conclusions

BTPOA is a promising CHON alternative to SO_3_-Ph-BTP for An/Ln separation in the *i*-SANEX system through solvent extraction. The results show the optimal HNO_3_ concentration range is 0.3–0.7 M, reaching the highest separation factors (SF_Eu/Am_ ∼ 120) in the TODGA system. However, BTPOA does not reach the same high values as its sulfonated analogue. The complexation reaction was fast, reaching equilibrium for An­(III) and light Ln­(III) in ca. 10 min. For heavy Ln­(III), equilibrium is reached within a time frame of 30–50 min. A remarkable stability against hydrolysis was found in low HNO_3_ concentrations (≤0.8 M) over a six-month period. At higher HNO_3_ concentrations (≥0.8 M), the observed results indicate slight changes in metal ion complexation that could be related to acid-induced hydrolysis.

The slope analysis conducted with solvent extraction yielded inconclusive data, whereas the TRLFS method revealed the formation of 1:3 complexes for Cm­(III) and Eu­(III) during extraction. Conditional stability constants for Cm­(III) in HClO_4_ indicate that BTPOA is a stronger ligand than its analogue SO_3_-Ph-BTP. However, in NO_3_^–^ media, lower conditional stability constants were found, though these were similar to those identified for SO_3_-Ph-BTP. The remarkably high FI factor exhibited by Eu­(III) hindered the determination of conditional stability constants using TRLFS.

The BTPOA molecule was also found to react with all four common reprocessing radical products from aqueous phase radiolysis—the e_aq_^–^, H^•^, ^•^OH, and NO_3_^•^. Notably, substitution of R-SO_3_H with R-COOH groups, in going from SO_3_-Ph-BTP to BTPOA, resulted in a significant reduction (by an order of magnitude) in reactivity toward NO_3_^•^. This finding is promising for the longevity and recyclability of a BTPOA-based separation solvent, as the rate of BTPOA radiolysis is expected to significantly decrease with increasing HNO_3_ concentration, which corresponds with an increasing prevalence of NO_3_^•^. However, additional studies are necessary to assess the effect of actinide complexation on the radiolytic longevity of BTPOA.^48^

## Supplementary Material



## Notes

Experimental details, materials, and equipment for the experiments; additional graphs from solvent extraction experiments; p*K*_a_ determination; TRLFS titration; pulse radiolysis experiments; and reaction kinetics figures for the reaction of BTPOA with the e_aq_^–^ and H^•^(PDF)
