# Supplementary material for: Extraction, Characterization, and Stability Studies of Bistriazinyl-Derived Carboxylic Acids
Source: Ind Eng Chem Res. 2026 Mar 13;65(11):6127–42. doi: 10.1021/acs.iecr.5c04766 (PMC13022820; doi:10.1021/acs.iecr.5c04766)
Supplement: Supplementary file 1 [file ie5c04766_si_001.pdf]

Extraction, characterization, and stability studies of Bistriazinyl-derived carboxylic acids

Laura Diaz Gomez[1], Patrik Weßling,[2], Andreas Wilden[1], Petra J. Panak[2], Gregory P. Horne[4], Stephen P. Mezyk[5], Julie R. Peller[6], Andreas Geist[3], and Giuseppe Modolo[1]*

1 Forschungszentrum Jülich GmbH, Institute of Fusion Energy and Nuclear Waste Management – Nuclear Waste Management (IFN-2), 52428 Jülich, Germany.

2 Heidelberg University, Institute of Physical Chemistry, Im Neuenheimer Feld 253, 69120 Heidelberg

3 Karlsruhe Institute of Technology (KIT), Institute for Nuclear Waste Disposal (INE), P.O. Box 3640, 76021 Karlsruhe, Germany.

4 Center for Radiation Chemistry Research, Idaho National Laboratory, P.O. Box 1625, Idaho Falls, ID83415, USA.

5 Department of Chemistry and Biochemistry, California State University Long Beach, 1250 Bellflower Boulevard, Long Beach California, 90840-9507, USA.

6 Department of Chemistry, 1710 Chapel Drive, Valparaiso University, Valparaiso, IN, 46383, USA.

*Corresponding author: a.wilden@fz-juelich.de

Table of Contents

Chemicals 2
Equipment 3
Solubility 4
Acid constant determination 5
Solvent Extraction 6
  Kinetics 6
  Simulated HAR 6
TRLFS 7
  Cm in $HClO_4$ 7
  Eu in HNO_3 9
Electron pulse irradiation experiments 10
  BTPOA + Hydrated Electron 12
  BTPOA + Hydrogen Atom 13
References 13

Chemicals

BTP-Octa carboxylic acid (BTPOA) >99%, BTBPOA >99%, BTPhenOA >99%, and TODGA 99% were purchased from Technocomm Ltd. Falkland, Scotland. Concentrated solutions of perchloric acid ($HClO_4$) p.a, and nitric acid (HNO_3) p.a were obtained from MERCK or Sigma-Aldrich, Darmstadt, Germany or USA. All chemicals were used without further purification. Radioactive tracers include 10 µM ^{241}Am, ^{244}Cm, and ^{152}Eu used for the extraction experiments. The HNO_3 solutions include all lanthanides excluding Pm in a concentration of 10 µM of each Ln(III). In TRLFS experiments, a stock solution of $^{248}Cm(ClO_4)_3$ (500 Bq, 21.2 µM) was used for Cm complexation and an $Eu(ClO_4)_3$ stock solution (1.07 mM) was used for Eu experiments. ISANE IP175 was obtained by CEA Marcoule. 1,2-Cyclohexylenedinitrilotetraacetic acid (CDTA) >99% was purchased from Sigma-Aldrich, Steinheim, Switzerland. All dilutions were made using ultra-pure water (18.2 MΩ cm).

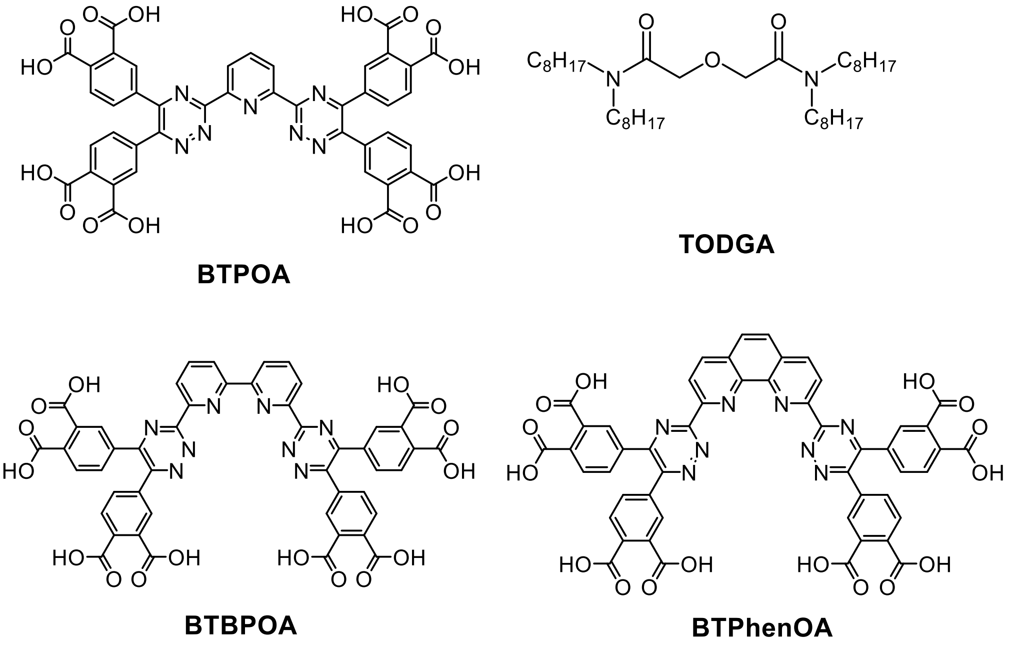

Figure S1. Molecular structures of the CHON analogs tested in the studies and the corresponding pair extractant.

Equipment

For pKa determination, a Titrando 907 automatic titrator was used, purchased from Metrohm GmbH & Co. KG (Filderstadt, Germany), calibrated with five buffer solutions of pH 1 ± 0.05 at 20 °C from Carl-Roth Karlsruhe Germany, potassium hydrogen phthalate pH 4 ± 0.01 at 25 °C potassium phosphate/disodium phosphate pH 7 ± 0.01 at 25 °C, sodium carbonate/sodium hydrogen carbonate pH 10 ± 0.02 at 25 °C from Carl-Roth Italy and disodium hydrogen phosphate/sodium hydroxide pH 12± 0.02 at 25 °C from Merck Darmstadt Germany and measuring with Metrohm glass electrodes (Unitrode with Pt1000 – high performance and Microelectrode). For UV-Vis/NIR measurements a Perkin Elmer UV-Vis Spectrometer Lambda 19 was used with High Precision Cell Quartz Glass light path 10 mm cuvettes from QS Helma Analytics.

For TRLFS studies all experiments were performed at 24.85°C with a Nd:YAG (Surelite II laser, Continuum) pumped dye laser system (NarrowScan D-R; Radiant Dyes Laser Accessories GmbH). The excitation wavelength for Cm(III) is 396.6 and 394 nm for Eu(III). A spectrograph (Shamrock 303i, ANDOR) with 1199 lines per mm gratings was used for spectral

deconvolution. The fluorescence emission was detected by an ICCD camera (iStar Gen III, ANDOR) after a delay time of 1 µs using a gate width of 1 ms to discriminate short-lived, organic fluorescence and light scattering.

Solubility

The solubility of the three hydrophilic ligands was tested against different concentrations of HNO_3. BTPOA is completely soluble in acidic media. On the other hand, the bipyridine (BTBPOA) and phenanthroline (BTPhenOA) analogues are soluble in water but not soluble in HNO_3 regardless of the concentration (**Figure S2**).

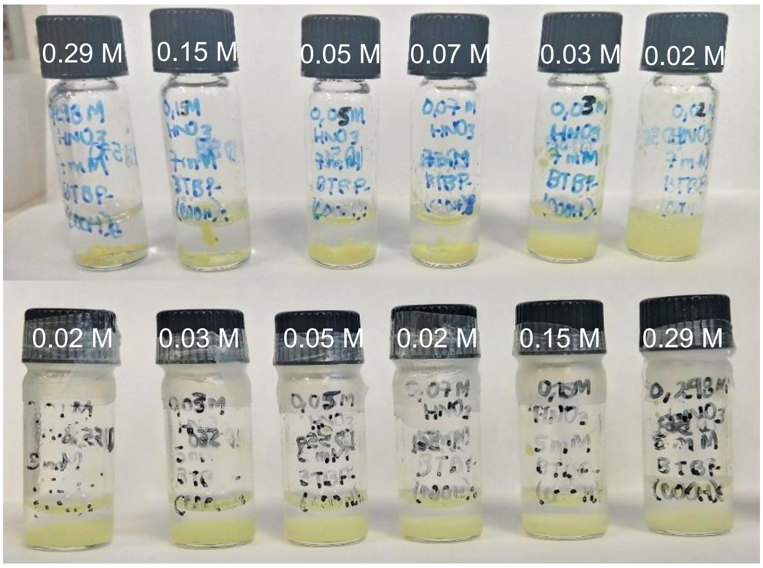

Figure S2. Solubility test of 5 mM BTBPOA at different HNO_3 concentrations. Top: before temperature. Bottom: after temperature water bath (45–70 °C).

Even though we seek to improve solubility at higher acidity enhanced through temperature (45–65 °C) in an ultrasonic bath, at the end the ligand slightly dissolves or remains the same (**Figure S2**). Whereas BTPhenOA solubility in acidic media reached the range between 0.01–0.05 M HNO_3. For higher HNO_3 concentrations, we also attempted to use an ultrasonic water bath for temperatures between 45–70 °C, in this case, the ligand was dissolved but after cooling down it creates a gel suspension which is not suitable for solvent extraction.

Acid constant determination

The first titration experiment was conducted in triplicate using both acid and base additions to assess reproducibility; the results are shown in **Figure S3**.

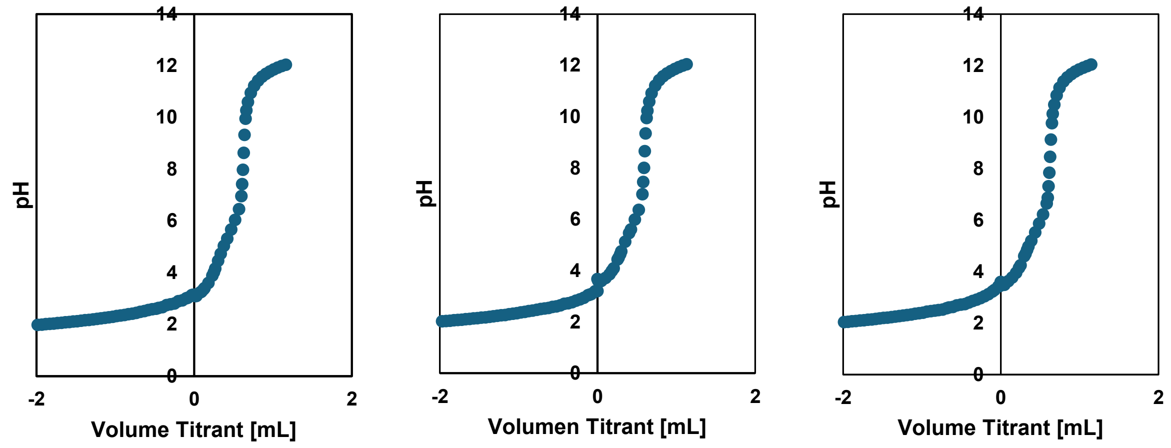

Figure S3. Potentiometric titration in triplicate with 0.1 M HNO_3 (to the left) and 0.1 M NaOH (to the right). Exp. Cond. 8 mg BTPOA diluted in H_2O at room temperature

Following this experiment, the titrated BTPOA concentration was increased, and the corresponding results are shown in **Figure S4**.

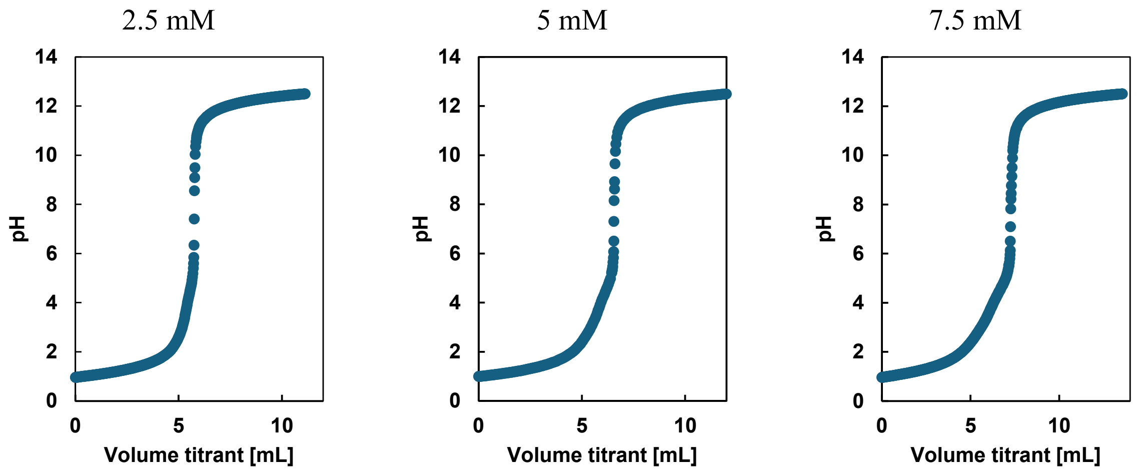

Figure S4. Potentiometric titration with 0.1 M NaOH + $NaNO_3$ at different ligand concentrations. Exp. Cond. 30 mg BTPOA diluted in 0.1 M HNO_3 + $NaNO_3$ at room temperature

In the case of BTBPOA and BTPhenOA, the total number of exchangeable protons (eight) was not reached; this can be attributed to the early ionization of the ligands. Considering the poor solubility of BTBPOA and BTPhenOA in acidic solution, the titration likely caused precipitation (unseen). Therefore, pK_a values for these two ligands are not reported.

Solvent Extraction

Kinetics

Figure S5 shows the comparison of the back extraction and normal extraction at 60 min.

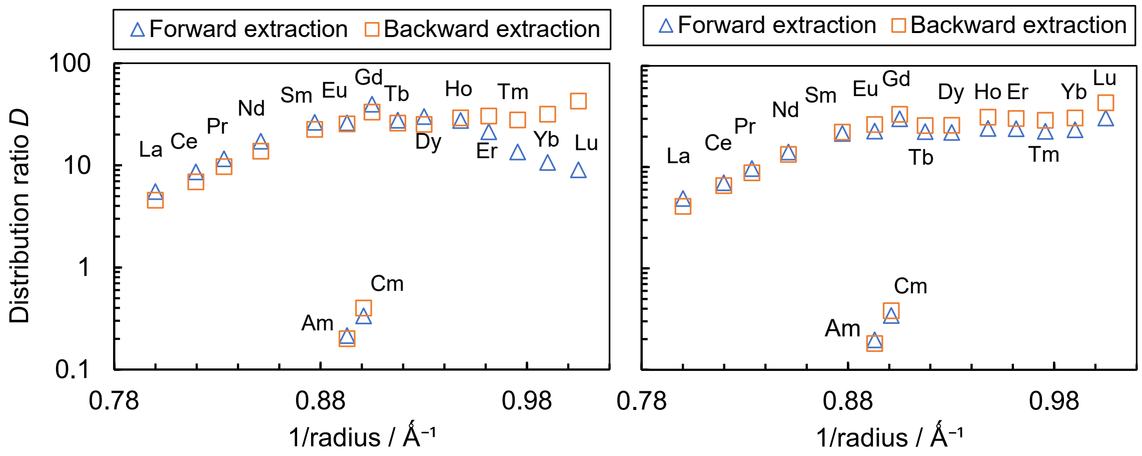

Figure S5. Kinetics comparison at equilibrium for forward and backward extraction as a function of inverse ionic radius. Left: 30 min. Right: 60 min. Exp. cond.: Org.: 0.2 M TODGA in 5% octanol/ISANE IP175. Aq.: 0.01 M BTPOA in 0.5 M HNO_3; 10 µM Ln(III) (w/o Pm), spiked with ^{241}Am, ^{244}Cm, and ^{152}Eu at 22 °C and O/A = 1.

Simulated HAR

According to Modolo and Sypula *et al.*[1, 2] adding CDTA to HAR as masking agent avoids Zr and Pd co-extraction. Otherwise, the metals ions can partially inhibit the BTPOA capability (**Figure S6**) reducing the separation factors between Eu and Am leading to precipitation and third phase formation. **Table S1** displays the composition and concentration of the simulated HAR solution used in this experiment.

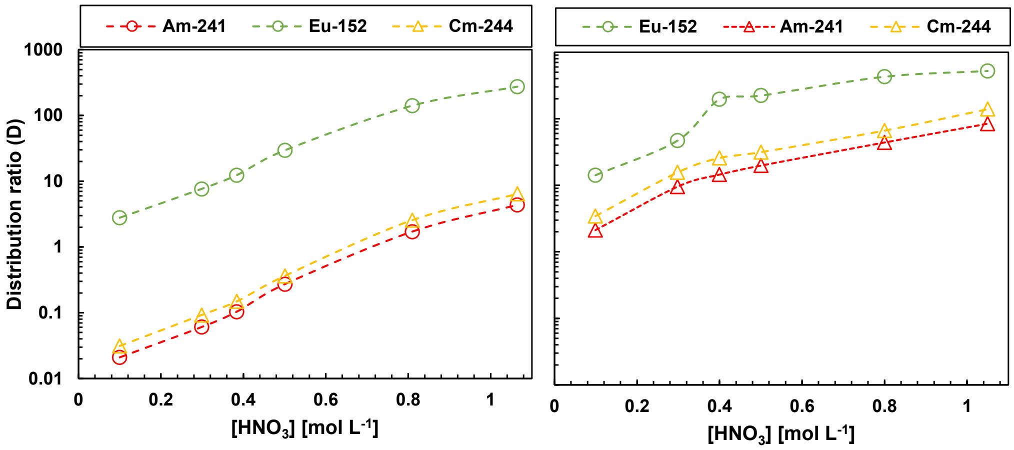

Figure S6. Comparison of HAR extraction with (left) and without (right) 0.05 M CDTA. Exp. cond: Aq.: HAR in 2.96 M HNO_3, scrubbing with 0.5 M HNO_3 and back-extracted with 0.01 M BTPOA in HNO_3. Org.: 0.2 M TODGA in 5% octanol/ISANE IP175 and O/A = 1.

Table S1. Composition and concentration of the simulated HAR solution.

Element	Concentration [mM]	Element	Concentration [mM]
Al	0.15	Ag	0.06
Cr	3.33	Cd	0.14
Fe	70.64	Sn	0.10
Ni	1.57	Sb	0.06
Cu	0.64	Te	0.79
Se	0.37	Cs	3.84
Rb	2.36	Ba	1.92
Sr	6.50	La	1.72
Y	3.42	Ce	4.19
Zr	21.40	Pr	1.55
Mo	23.30	Nd	4.95
Ru	18.49	Sm	1.00
Rh	3.21	Eu	0.21
Pd	13.19	Gd	0.15

TRLFS

Cm in $HClO_4$

The fraction of each species was determined by peak deconvolution of the Cm(III) fluorescence spectra (**Figure S7**) starting by subtracting the known $[Cm(H_2O)_9]^{3+}$ spectrum from the measured spectra to determine the fluorescence spectrum of the 1:1 complex. This is

further used to derive the fluorescence spectrum of the 1:2 complex and so on. Single component spectra for each species were determined and are shown in **Figure S7**. **Figure S8** shows the slope analyses which confirm that one ligand molecule is added at each complexation step.

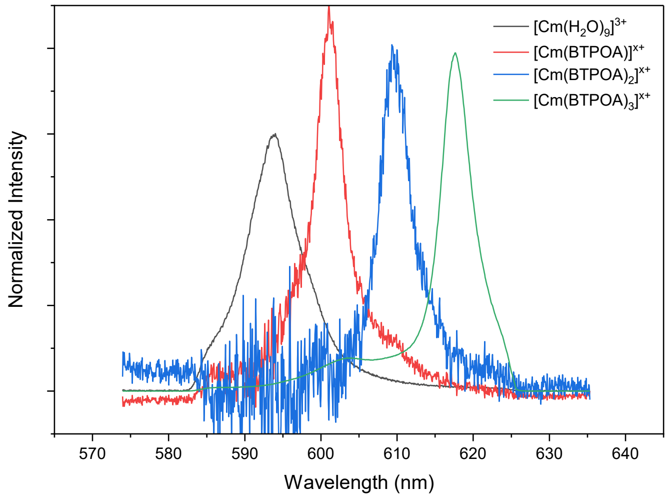

Figure S7. Single component spectra of the $[Cm(BTPOA)_n]$ complexes (n = 1–3) in 1 mM $HClO_4$.

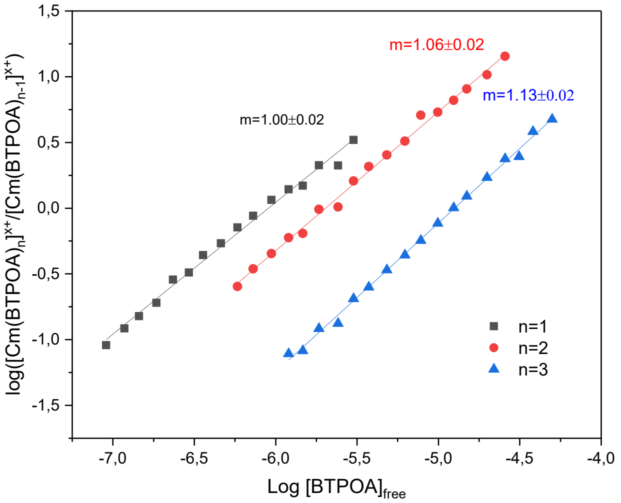

Figure S8. Slope analyses for the complexation of Cm(III) with BTPOA in 1 mM $HClO_4$. Double logarithmic plot of the concentration ratios $[Cm(BTPOA)_n]/[Cm(BTPOA)_{n-1}]$ as a function of the non-complexed ligand concentration.

Fluorescence lifetime measurements are generally used to determine the number of ligand and water molecules using the Kimura equation.[3] **Figure S9** shows the decrease of the fluorescence intensity as a function of the delay time for $[Cm(H_2O)_9]^{3+}$ and $[Cm(BTPOA)_3]^{x+}$. 67 µs corresponds to 9 water molecules in the inner coordination sphere of the Cm(III) aquo species, whereas 151 µs would relate to four H_2O molecules for the 1:3 Cm-BTPOA complex

instead of 3 H_2O molecules as should be expected for BTP complexes. These results confirm that the aromatic moieties of the ligand quench the fluorescence of Cm(III).

As demonstrated in the relevant literature, it is possible for molecules to engage in dynamic quenching through collusion with fluorophores, thereby producing the phenomenon of quenching[4-6]. It can thus be concluded that the Kimura equation is not applicable to this system.

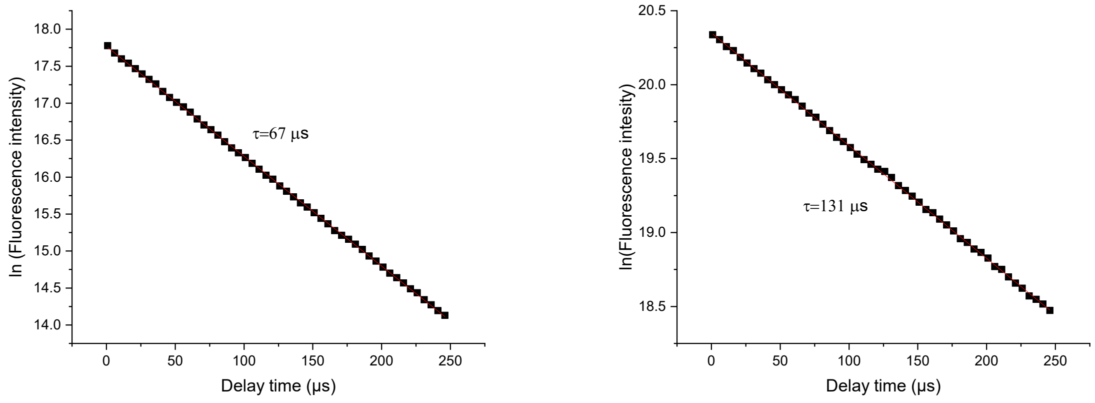

Figure S9. Decrease of the fluorescence intensity as a function of the delay time for $[Cm(H_2O)_9]^{3+}$ (left) and $[Cm(BTPOA)_3]^{x+}$ (right).

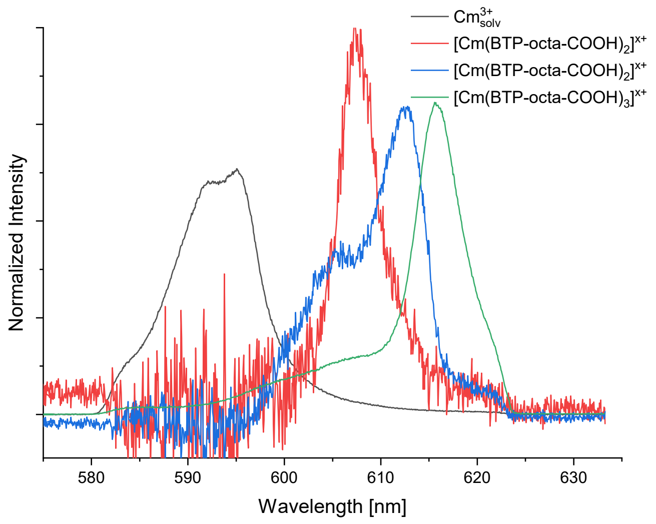

Figure S10. Normalized fluorescence spectra of [Cm(BTPOA)n] complexes in 0.5 M HNO_3.

Eu in HNO_3

The stoichiometry of the different Eu(III) BTPOA complexes is confirmed by slope analyses according to a stepwise complexation model. Plotting the logarithm of

([Eu(BTPOA)$_n$]$^{x+}$/Eu(BTPOA)$_{n-1}$]$^{x+}$) as a function of the logarithm of the non-complexed BTPOA concentration (see **Figure S11**) results in slopes of 1 for each complex species (n = 1, 2, or 3).

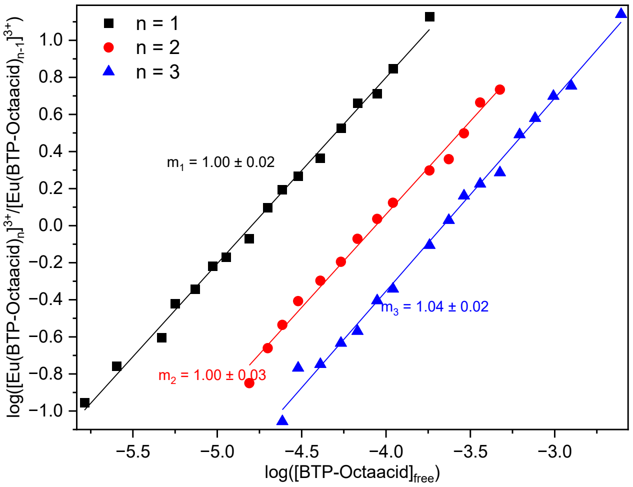

Figure S11. Slope analyses for the complexation of Eu(III) with BTPOA in 0.5 M HNO_3. Double logarithmic plot of the concentration ratios [Eu(BTPOA)n]/[Eu(BTPOA)$_{n-1}$] as a function of the concentration of non-complexed ligand.

Electron pulse irradiation experiments

Chemical kinetics were determined for the reaction of 2,6-bis [5,6-di(3,4-dicarboxyphenyl)-1,2,4-trazin-3-yl] pyridine (BTPOA) with radical products (e_{aq}^-, H$^•$, $^•$OH, and $NO_3^•$) arising from the radiolysis of dilute to concentrated HNO_3. Transient reaction kinetics were measured using the Notre Dame Radiation Laboratory (NDRL) pico-to-nanosecond electron pulsed linear accelerator (LINAC)/transient absorption detection facility.[7,8] Irradiation samples comprised of BTPOA (>99%, Technocomm Ltd.) dissolved in aqueous solutions designed to isolate the reaction kinetics of specific radicals.

- *Hydrated Electron (e_{aq}^-).* Nitrogen gas (N_2, 99.5%, Airgas) saturated aqueous solutions of 0.5 M tertiary butanol (*t*BuOH, >99.5%, MilliporeSigma) and 10 mM phosphate (>99%, MilliporeSigma) buffer at pH 7.0 ± 0.1, as adjusted by perchloric acid ($HClO_4$, 70%, MilliporeSigma) and sodium hydroxide (NaOH, Fisher, >99.5%, MilliporeSigma):

$^{\bullet}OH + tBuOH \rightarrow [tBuOH(-H)]^{\bullet} + H_2O$ $\quad k = 6.0 \times 10^8\ M^{-1}\ s^{-1}$,[9] (S1)

$H^{\bullet} + tBuOH \rightarrow [tBuOH(-H)]^{\bullet} + H_2$ $\quad k = 1.7 \times 10^5\ M^{-1}\ s^{-1}$.[9] (S2)

Decay of the isolated e_{aq}^{-} was directly observed at 720 nm.

- *Hydrogen Atom ($H^{\bullet}$)*. N_2 saturated aqueous solutions of 20 mM tertiary tBuOH and 10 mM phosphate buffer at pH 2.0 ± 0.1, as adjusted by $HClO_4$ and NaOH:

$e_{aq}^{-} + H_{aq}^{+} \rightarrow H^{\bullet}$ $\quad k = 2.3 \times 10^{10}\ M^{-1}\ s^{-1}$.[9] (S3)

Growth kinetics of the transient BTPOA adduct, $[BTPOA(+H)]^{\bullet}$, were directly monitored at its peak absorption determined here to be at 380 nm:

$H^{\bullet} + BTPOA \rightarrow [BTPOA(+H)]^{\bullet}$. (S4)

- *Hydroxyl Radical ($^{\bullet}OH$)*. Nitrous oxide gas (N_2O, 99%, Airgas) saturated aqueous solutions of 10 mM phosphate buffer at pH 7.0 ± 0.1, as adjusted by $HClO_4$ and NaOH:

$e_{aq}^{-} + N_2O \rightarrow {}^{\bullet}O^{-} + N_2$ $\quad k = 9.1 \times 10^9\ M^{-1}\ s^{-1}$,[9] (S5)

${}^{\bullet}O^{-} + H_2O \rightleftharpoons {}^{\bullet}OH + OH^{-}$ $\quad pKa = 11.84$,[10] (S6)

$H^{\bullet} + N_2O \rightarrow {}^{\bullet}OH + N_2$ $\quad k = 2.1 \times 10^6\ M^{-1}\ s^{-1}$.[11] (S7)

Growth kinetics of the transient BTPOA adduct, $[BTPOA(-H)]^{\bullet}$, were directly monitored at its peak absorption determined here to be at 410 nm:

${}^{\bullet}OH + BTPOA \rightarrow [BTPOA(+OH)]^{\bullet}$. (S8)

- *Nitrate Radical ($NO_3^{\bullet}$)*. N_2O saturated aqueous solutions of 6.0 M HNO_3 (≥99.999% Trace Metals Basis, MilliporeSigma) promote the formation of $NO_3^{\bullet}$. Decay of the isolated $NO_3^{\bullet}$ was directly observed at 630 nm. Note, growth of the corresponding adduct:

$NO_3^{\bullet} + BTPOA \rightarrow [BTPOA(+NO_3)]^{\bullet}$, (S9)

was not monitored directly due to the $NO_3^{\bullet}$ radical's large absorption band.

All transient absorption measurements were made using a 1.0 cm optical path length quartz flow-cell, with flow rate and temperature regulated to ensure that each electron pulse irradiated a fresh sample. Solution temperatures were directly measured by an in-flow thermocouple placed immediately above the irradiation cell except for the HNO_3 solutions. The temperature stability of the system was better than ± 0.3 °C. Kinetic traces were generated through averaging 8−16 individual measurements. LINAC dosimetry was established each day using N_2O saturated aqueous solutions of 10 mM potassium thiocyanate (KSCN, ≥99.0% ACS Reagent Grade, MilliporeSigma) at λ_{max} = 472 nm ($G\varepsilon = 5.2 \times 10^{-4}$ m^2 J^{-1}).[12] Ultra-pure water (≥ 18.2 MΩcm), sourced from NDRL, was used in the preparation of all aqueous solutions.

BTPOA + Hydrated Electron

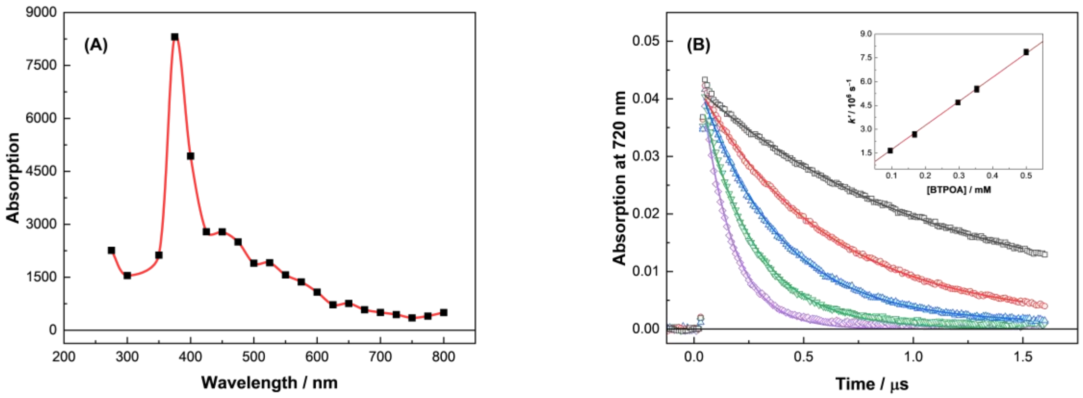

Figure S12. (A) Transient absorption spectra for reduced BTPOA observed at 1.0 μs after the electron pulse irradiation of 500 μM BTPOA in N_2 saturated aqueous solution of 0.5 M tBuOH and 10 mM phosphate buffer (pH 7.0 ± 0.1) at 24.6 °C. **(B)** Corresponding first-order transient decay kinetics observed at shorter-times at 720 nm for 0 (**grey**), 95.7 (**red**), 168 (**blue**), 297 (**green**), and 500 (**purple**) μM BTPOA. *Inset:* Second-order rate coefficient determination using the fitted pseudo first-order rate coefficient values: (2.79 ± 0.05) × 10^5, (1.63 ± 0.01) × 10^6, (2.68 ± 0.01) × 10^6, (5.59 ± 0.03) × 10^6, and (7.83 ± 0.04) × 10^6 s^{-1}, respectively. Solid line is a weighted linear fit to transformed data, corresponding to k(BTPOA + e_{aq}^-) = (1.60 ± 0.02) × 10^{10} M^{-1} s^{-1}, R^2 = 0.999.

BTPOA + Hydrogen Atom

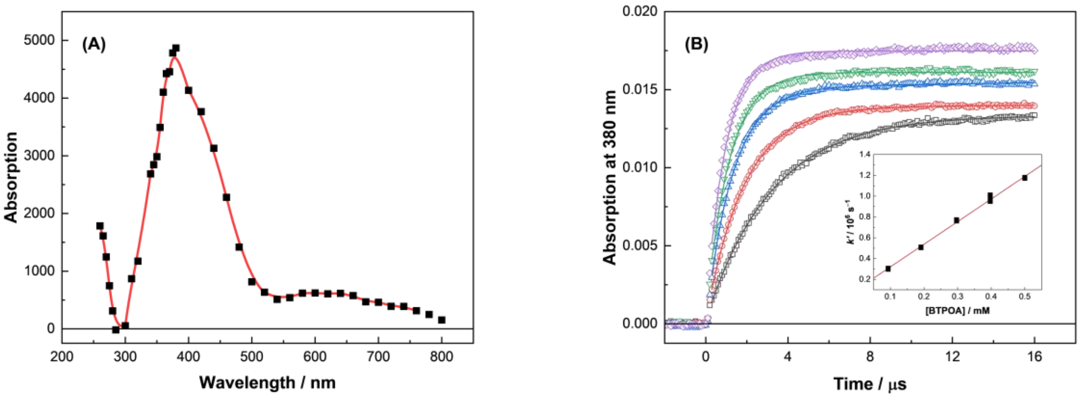

Figure S13. (A) Transient absorption spectra arising from the electron pulse irradiation of 500 μM BTPOA in N_2 saturated aqueous solution of 20 mM tBuOH and 10 mM phosphate buffer (pH 2.0 ± 0.1) at 24.7 °C. **(B)** Corresponding first-order transient growth kinetics observed at 380 nm for 95.3 (**grey**), 191 (**red**), 297 (**blue**), 397 (**green**), and 500 (**purple**) μM BTPOA. *Inset:* Second-order rate coefficient determination using the fitted pseudo first-order rate coefficient values: of $(2.97 \pm 0.02) \times 10^5$, $(5.10 \pm 0.05) \times 10^5$, $(7.73 \pm 0.06) \times 10^5$, $(9.64 \pm 0.09) \times 10^5$, and $(1.18 \pm 0.01) \times 10^6$ s^{-1}, respectively. Solid line is a weighted linear fit to transformed data, corresponding to $k(\mathrm{BTPOA} + \mathrm{H}^\bullet) = (2.17 \pm 0.03) \times 10^9$ M^{-1} s^{-1}, $R^2 = 0.997$.

References

(1) Modolo, G.; Wilden, A.; Kaufholz, P.; Bosbach, D.; Geist, A. Development and demonstration of innovative partitioning processes (i-SANEX and 1-cycle SANEX) for actinide partitioning. *Progr. Nucl. Energ.* **2014**, *72*, 107-114. DOI: 10.1016/j.pnucene.2013.07.021 (acccessed 2013-09-19).

(2) Sypula, M.; Wilden, A.; Modolo, G.; Geist, A. Innovative SANEX process for actinide(III) separation from PUREX raffinate using TODGA-based solvents. In *19th International Solvent Extraction Conference*, Santiago de Chile, Chile, 3-7 October, 2011; L., F. V., Moyer, B. A., Eds.; Gecamin Ltda. Paseo Bulnes 197, Piso 6, Santiago, Chile, Postal Code: 833 0336: 2011; p P93.

(3) Kimura, T.; Choppin, G. R.; Kato, Y.; Yoshida, Z. Determination of the hydration number of Cm(III) in various aqueous solutions. *Radiochimica Acta* **1996**, *72* (2), 61-64, Article.

(4) Weigl, M.; Denecke, M. A.; Panak, P. J.; Geist, A.; Gompper, K. EXAFS and time-resolved laser fluorescence spectroscopy (TRLFS) investigations of the structure of Cm(III)/Eu(III) complexed with di(chlorophenyl)dithiophosphinic acid and different

synergistic agents. *Dalton Transactions* **2005**, (7), 1281-1286, Article. DOI: 10.1039/b418371a (acccessed 2013-08-08).

(5) Yun, C. Y.; Ryu, K. H.; Lee, C.; Lee, G. G.; Jo, S.; Kim, K. H.; Sung, S. W. Modeling and Simulation of Solvent Extraction Processes for Purifying Rare Earth Metals Using Saponified PC88A. *Journal of Chemical Engineering of Japan* **2017**, *50* (9), 716-726, Article. DOI: 10.1252/jcej.15we058 (acccessed 2023-02-02).

(6) Stumpf, S.; Billard, I.; Panak, P. J. Solution Chemistry of Cm(III) and Eu(III) in Ionic Liquids. In *Ionic Liquids Iv: Not Just Solvents Anymore*, Brennecke, J. F., Rogers, R. D., Seddon, K. R. Eds.; Acs Symposium Series, Vol. 975; Amer Chemical Soc, 2007; pp 247-256.

(7) Whitman, K.; Lyons, S.; Miller, R.; Nett, D.; Treas, P.; Zante, A.; Fessenden, R. W.; Thomas, M. D.; Wang, Y. Linear accelerator for radiation chemistry research at Notre Dame 1995. In *'95 Particle Accelerator Conference & International Conference of High Energy Accelerators*, Dallas, TX, USA, 1996.

(8) Hug, G. L.; Wang, Y. C.; Schoneich, C.; Jiang, P. Y.; Fessenden, R. W. Multiple time scales in pulse radiolysis. Application to bromide solutions and dipeptides. *Radiat. Phys. Chem.* **1999**, *54* (6), 559-566, Article. DOI: 10.1016/s0969-806x(98)00303-x (acccessed 2019-05-13).

(9) Buxton, G. V.; Greenstock, C. L.; Helman, W. P.; Ross, A. B. Critical Review of Rate Constants for Reactions of Hydrated Electrons, Hydrogen Atoms and Hydroxyl Radicals ($^{\cdot}OH/^{\cdot}O^{-}$) in Aqueous Solution. *Journal of Physical and Chemical Reference Data* **1988**, *17* (2), 513-886, Review. DOI: 10.1063/1.555805 (acccessed 2015-05-13).

(10) Mezyk, S. P. Determination of the rate constant for the reaction of hydroxyl and oxide radicals with cysteine in aqueous solution. *Radiat. Res.* **1996**, *145* (1), 102-106, Article. DOI: 10.2307/3579203.

(11) Czapski, G.; Peled, E. ON PH-DEPENDENCE OF GREDUCING IN RADIATION CHEMISTRY OF AQUEOUS SOLUTIONS. *Isr. J. Chem.* **1968**, *6* (4), 421-&, Article.

(12) Buxton, G. V.; Stuart, C. R. Reevaluation of the Thiocyanate Dosimeter for Pulse-Radiolysis. *Journal of the Chemical Society-Faraday Transactions* **1995**, *91* (2), 279-281, Article. DOI: 10.1039/ft9959100279 (acccessed 2019-05-13).
